# PhyGeo-KG: Physics-Regularized Distant Supervision for Multimodal Geometric Knowledge Graph Construction in Catenary Maintenance

**DOI:** 10.3390/s26072155

**Published:** 2026-03-31

**Authors:** Tianguo Jin, Xinglong Chen, Dongliang Zhang, Bingxiang Zeng

**Affiliations:** School of Mechatronics Engineering, Harbin Institute of Technology, Harbin 150001, China; jintg@hit.edu.cn (T.J.); 15990144125@163.com (D.Z.); 17348191277@163.com (B.Z.)

**Keywords:** knowledge graph construction, distant supervision, physics regularization, catenary maintenance, building information modeling (BIM)

## Abstract

High-speed railway catenary maintenance increasingly requires knowledge bases that can connect maintenance records with geometric infrastructure models for reliable digital twin-enabled decision support. However, existing knowledge graph construction methods in engineering settings often struggle with severe label sparsity, weak instance-level grounding, and limited physical interpretability. To address these issues, we propose PhyGeo-KG, a physics-regularized distant supervision framework for constructing high-fidelity multimodal geometric knowledge graphs for catenary maintenance. The framework consists of three main phases: (i) a Semantic–Geometric–Physical–Procedural ontology for unifying heterogeneous engineering information; (ii) a deterministic grounding strategy that aligns textual mentions with Industry Foundation Classes (IFC)/Building Information Modeling (BIM) entities through geometric interfaces; and (iii) a physics-aware refinement and ontology-driven evolution process that removes physically implausible relations while expanding the validated graph. Experiments on a real-world dataset constructed from IFC-compliant BIM models of a 2 km high-speed railway catenary section and associated maintenance documents show that the proposed approach improves relation precision and physical consistency while effectively suppressing semantic hallucinations. A case study further demonstrates its potential for instance-level fault localization in semantic digital twins. These results indicate that PhyGeo-KG provides an interpretable and transferable foundation for physics-regularized multimodal geometric knowledge graph construction and digital-twin-enabled decision support in catenary maintenance, with potential to support future sensor-integrated maintenance applications.

## 1. Introduction

High-speed railway catenary systems serve as the primary power conduit for electrified rail transport, operating in heterogeneous outdoor settings where reliability directly determines operational safety and service efficiency. With the advancement of Industry 4.0 and Digital Twin (DT) technologies, maintenance practice is moving from periodic manual inspections toward intelligent, data-driven condition-based maintenance (CBM). This shift produces large volumes of heterogeneous data, dominated by unstructured maintenance logs that document historical failures, procedures, and expert experience, together with structured Building Information Modeling (BIM) assets encoded in Industry Foundation Classes (IFC) that encode high-fidelity geometric and topological information. Semantic Digital Twin (SDT) research also underscores the need for interoperable, machine-interpretable representations that connect these data assets to physical infrastructure for cross-system integration and lifecycle management [1,2,3,4,5]. However, converting fragmented records into actionable knowledge remains challenging: textual narratives convey “what happened,” whereas BIM/IFC models specify “where it is,” thereby creating a semantic–geometric disconnect that hinders the construction of high-fidelity engineering knowledge graphs (KGs).

Knowledge graphs (KGs) have become a cornerstone for organizing and reasoning over multi-source heterogeneous data in complex engineering systems [6,7,8]. Domain-specific KGs have shown utility across industrial sectors, including supply-chain management [9], safety risk identification [10], and fault diagnosis for intelligent production lines [11]. In railway-related contexts, ontologies and KGs have been investigated to support safety-oriented knowledge organization and maintenance-centric decision making [12,13]. In parallel, SDT studies emphasize ontology-driven interoperability between semantic descriptions and physical assets, seeking to align data assets and enable consistent interpretation across tools and stakeholders [1,2,3,4,5]. Within digital twin railway research, integrated representations that couple geospatial data, models, and knowledge further motivate instance-level linkage between operational records and spatially grounded infrastructure elements [14].

To connect abstract semantics with physical reality, recent approaches have increasingly integrated geometric data from Computer-Aided Design (CAD) and BIM sources. Representative advances include constructing KGs from large CAD repositories [15], performing geometry-informed feature recognition from manufacturing models [16], and enriching BIM semantics via geometric relation checking [17]. Semantic–geometric representations have also been developed for complex 3D modeling scenarios [18]. At the methodological level, engineering informatics has also drawn on structured design theories to model functional and physical relationships, including the Function–Behavior–Structure (FBS) framework [19,20] and Product–Process–Resource (PPR)-style paradigms [21,22]. Meanwhile, for label-sparse domains where expert annotation is expensive, distant supervision (DS) has become a dominant paradigm for relation extraction by generating weak labels through alignment between text mentions and existing knowledge bases; Snorkel provides a representative weak-supervision framework for rapid label creation and aggregation [23]. Subsequent DS research has employed denoising strategies—such as attention mechanisms and reinforcement learning—to mitigate inherent labeling noise [24,25]. Complementing these efforts, physics-based simulation ontologies have been proposed to formalize physical quantities and constraints to support modeling consistency and reusability [26].

Despite these advancements, existing KG construction paradigms in engineering domains often remain “semantically rich but physically agnostic,” producing relations that are detached from geometric reality and governing physical constraints. In the specific context of catenary maintenance, this semantic–physical disconnect arises from three interrelated challenges. First, cross-modal heterogeneity creates a grounding gap: maintenance logs describe components and operations in context-dependent linguistic terms, whereas BIM/IFC models encode precise geometry and topology that are not directly indexed by those terms [14,17]. Consequently, the extracted relations may lack reliable instance-level traceability to specific physical entities, limiting their value for SDT workflows that require localization and verification. Second, the domain suffers from severe label sparsity and cold-start conditions, making fully supervised relation extraction impractical. Although distant supervision (DS) offers a practical alternative, it inherently introduces systematic noise. Purely NLP-based models tend to over-emphasize co-mention patterns, leading to a “long-distance semantic bias” in which relations that are linguistically plausible but spatially or topologically infeasible receive high confidence scores [25]. Third, geometry is often treated as static metadata or applied only as a post hoc filter, rather than as an active, computable regularizer embedded throughout the extraction-and-refinement loop. As a result, unified and interpretable mechanisms for injecting explicit physical-feasibility cues—such as geometric adjacency and topological connectivity—into DS-driven KG construction remain limited [7,17].

To mitigate these issues, this work introduces PhyGeo-KG, a physics-regularized distant supervision framework for constructing high-fidelity multimodal geometric KGs for catenary maintenance. Following a white-box engineering instantiation philosophy (i.e., explicit, interpretable mechanism operators rather than black-box neural embeddings), the proposed approach makes three core contributions:

1. Multidimensional ontology for unified and computable representation. We construct a Semantic–Geometric–Physical–Procedural (S–G–P–P) four-dimensional ontology to unify heterogeneous engineering modalities. Beyond static taxonomies, it introduces computable mechanism operators and geometric interfaces (IFaces) as executable hooks, enabling consistent representation and reasoning across semantics, geometry, physics, and procedures.
2. Geometry-anchored deterministic grounding paradigm. To address zero-shot alignment under severe label sparsity, we propose a structure-aware deterministic grounding strategy. We parse BIM assets into a native IFC graph and leverage IFace anchors with ROI-based locality constraints to establish instance-level traceability between text mentions and BIM entities, providing an interpretable cold-start solution.
3. Physics-regularized distant supervision with dual semantic–geometric gating. We incorporate physical feasibility into the extraction-and-refinement loop via a dual semantic–geometric gating mechanism with a pluggable physics critic. By treating computable physical constraints as active regularizers, the proposed approach systematically suppresses semantically plausible yet physically infeasible relations and supports a prune-and-evolve refinement for reliable engineering KG construction.

The remainder of this paper is organized as follows: Section 2 reviews related work; Section 3 presents the problem formulation and framework overview; Section 4 details the methodology; Section 5 presents the evaluation protocol and case studies; Section 6 discusses the efficacy and boundaries of the proposed approach; and Section 7 concludes the paper and outlines future research directions.

## 2. Related Work

### 2.1. Domain-Specific Knowledge Graphs in Engineering Systems

Knowledge graphs (KGs) represent a powerful paradigm for integrating heterogeneous data and supporting intelligent decision-making in complex engineering environments [6,7]. In contrast to general-domain KGs, engineering KGs must adhere to industry standards, expert logic, and physical constraints to ensure reliability in safety-critical applications [6,13].

Representative applications include supply-chain management [9], safety risk identification in construction [10], and fault diagnosis in intelligent production lines [11,24]. In the railway domain, ontologies and KGs have been developed for emergency fault modeling [12], safety knowledge organization [13], and maintenance scheduling [27]. Collectively, these studies demonstrate the value of structured knowledge representations for maintenance-centric operations.

Of direct relevance to operational planning, Jin et al. [28] proposed a business-model-driven approach to task-planning KG construction that links high-level business logic with executable workflows. Extending this line of research, Jin et al. [29] introduced a discrete task decomposition method guided by knowledge graphs to optimize complex engineering scheduling. While these works effectively address procedural and task-level organization, they primarily operate at the semantic and functional levels. A key gap remains in linking these abstract task nodes to concrete geometric instances and explicitly tackling the upstream challenge of constructing physics-consistent relations from unstructured logs and BIM geometry.

### 2.2. Semantic Digital Twin and Geometric–Semantic Integration

Semantic Digital Twin (SDT) seeks to create machine-interpretable representations that bridge heterogeneous data assets with physical infrastructure, enabling interoperability and lifecycle management [1,2,3,4,5]. Recent SDT studies in construction [1], railway systems [14], and industrial environments [4] highlight the importance of ontology-driven alignment between textual records and geometric models.

To realize such alignment, researchers have increasingly incorporated geometric data from CAD and BIM sources into KGs. Bharadwaj and Starly [15] constructed product-design KGs from large CAD repositories using geometric similarity embeddings. Lilis et al. [17] advanced BIM semantic enrichment through automated geometric relation checking. Semantic 3D modeling of complex structures has also been explored by explicitly linking geometry and concepts [18]. Complementary ontological foundations, including the Function–Behavior–Structure (FBS) framework [19,20] and Product–Process–Resource (PPR) paradigm [21,22], provide theoretical support for representing functional and physical relationships [26]. In addition, standards such as STEP and OntoSTEP [30] facilitate the semantic interoperability of product data.

Beyond foundational alignment, recent SDT research increasingly emphasizes dynamic monitoring and semantic reasoning. For instance, comprehensive reference architectures and ontology frameworks have been proposed for Digital Twin Construction (DTC) to enable automated comparisons between project intent and actual execution [31]. In civil infrastructure, operational modal analysis is being integrated into digital twins to continuously update the structural health status of bridges [32]. Furthermore, advanced semantic digital twins now incorporate rule-based reasoning (e.g., SWRL and SHACL) to fuse IoT sensor data with BIM, automating safety compliance checking and hazard localization on construction sites [33].

Even with these advances, geometry is still largely treated as static metadata or used only as a post hoc filter. Few frameworks embed explicit geometric and topological feasibility constraints (e.g., adjacency and connectivity) as first-class, computable regularizers throughout the KG construction loop, thereby contributing to the “semantic–physical disconnect” described in this study.

### 2.3. Distant Supervision and Weak Supervision for Relation Extraction

In label-scarce engineering domains, Distant Supervision (DS) has become the dominant approach for relation extraction by automatically producing weak labels through alignment with existing knowledge bases [23]. The Snorkel framework [23] formalized weak supervision via labeling functions and generative models for rapid training data creation. Later studies have emphasized denoising strategies to mitigate the inherent “wrong labeling” noise, including attention mechanisms [25] and reinforcement learning to iteratively filter false positives [24]. Additionally, virtual knowledge graph construction methods have been explored to address zero-shot retrieval challenges in domain-specific contexts [34].

These techniques have been successfully applied to supply-chain KG construction [9] and fault diagnosis under Industry 4.0 [24]. Yet most DS pipelines remain language-driven and are vulnerable to systematic biases: models often assign high confidence to linguistically plausible but geometrically or topologically infeasible relations (the so-called “long-distance semantic bias”). Existing denoising strategies can mitigate semantic noise but often fail to suppress physically infeasible relations. This gap motivates incorporating explicit geometric and topological feasibility cues into the DS-driven refinement loop, ensuring that extracted relations are both semantically plausible and geometrically consistent.

## 3. Problem Formulation and Framework Overview

Catenary systems serve as the critical power artery for high-speed railways, necessitating a transition from manual inspection to intelligent, data-driven maintenance. A core challenge in this transition is achieving an integrated expression of semantic, geometric, physical, and procedural dimensions—the four pillars of catenary engineering information—to support reliable decision-making. However, constructing such a multi-dimensional knowledge base is hindered by the heterogeneity gap between unstructured logs and structured BIM geometry, label sparsity in failure records, and stringent traceability requirements.

To bridge these gaps, we propose the PhyGeo-KG framework. We formulate the construction process as a Physics-Regularized Distant Supervision problem, wherein physical laws act as explicit constraints to rectify semantic extraction noise. This chapter sets out the theoretical foundation for PhyGeo-KG, outlining the white-box design philosophy (Section 3.1) and the mathematical problem definition (Section 3.2), followed by a high-level overview (Section 3.3) that serves as the blueprint for the algorithmic implementation in Section 4.

### 3.1. Design Philosophy

To balance theoretical generality with engineering applicability in safety-critical domains, we present the methodology in two layers:

1. The General Framework: Mathematically defines how semantics, geometry, and physics are integrated into a unified regularization objective. At this level, the discriminative function and physical critic are defined as abstract operators, compatible with various implementations (e.g., neural networks or logical solvers).
2. The White-box Engineering Instantiation: Specifically targets severe label sparsity and stringent traceability requirements of the catenary maintenance domain. In this study, we instantiate the framework using lexicon-guided aggregators and explicit geometric solvers rather than opaque black-box neural networks. This strategic choice ensures that the extraction process is robust to cold-start conditions and fully interpretable for engineering validation.

### 3.2. Problem Formulation

The system takes two primary inputs: (1) geometric data ($D_{\mathrm{geo}}$), i.e., IFC-compliant BIM models of the catenary system; and (2) maintenance texts ($D_{\mathrm{txt}}$), i.e., large-scale unlabeled maintenance logs and regulations.

The framework produces a high-fidelity knowledge graph, denoted as $G^{*}$. To balance engineering rigor with knowledge coverage, the output is formalized as a two-stage deliverable: (i) a refined core graph ($G_{\mathrm{core}}^{*}$), which is a high-precision subset strictly regularized by the Physics Critic, serving as the trusted seed; and (ii) the final evolved graph ($G^{*}$), which is the expanded network derived from $G_{\mathrm{core}}^{*}$ via ontology-driven inference. Both graphs are required to be semantically valid and physically consistent, satisfying the domain-specific constraints defined in Γ.

The core engineering goal of PhyGeo-KG is to construct a high-fidelity multimodal geometric knowledge graph by selecting a physically plausible and logically valid subset of relations from the candidate edge set $E^{\mathrm{cand}}$ generated by deterministic grounding. This can be formulated as a discrete edge-selection problem, as expressed in Equation (1):

(1) $$E_{\mathrm{core}}^{*}=\arg\underset{E\subseteq E^{\mathrm{cand}}}{\max}\sum_{e\in E}(s_{e}-\beta v_{\mathrm{phy}}(e))\;s.t.\;E\;\mathrm{satisfies}\;\Gamma$$

where $s_{e}\in (0,1)$ is the semantic confidence score of the candidate edge e, $v_{\mathrm{phy}}(e)$ represents the explicit physical violation penalty, β is a hyperparameter balancing semantic recall and physical stringency, and Γ is the hierarchical constraint set.

Due to severe label sparsity and zero-shot conditions in the catenary maintenance domain, directly solving Equation (1) is infeasible. We therefore introduce a discriminative scorer $f_{\theta }$ to calibrate the semantic confidence $s_{e}$. Unlike opaque deep encoders, $f_{\theta }$ is explicitly restricted to an interpretable white-box model (instantiated as a linear aggregator with θ={w,b} in Section 4.3) and is optimized through the physics-regularized surrogate loss (Equation (9)). By minimizing this surrogate objective, $f_{\theta }$ is calibrated to suppress semantic hallucinations and align $s_{e}$ with physical plausibility. The refined core graph $G_{\mathrm{core}}^{*}$ is then constructed through dual semantic–geometric gating (Equation (10)), followed by post hoc constraint filtering using Γ, as detailed in Section 4.3.

To formalize the desired trade-off between semantic evidence and physical plausibility, we further introduce the physics-regularized surrogate objective in Equation (9). This objective should be interpreted as the general regularized formulation of the PhyGeo-KG scoring problem, while the current prototype operationalizes this principle through a deterministic white-box proxy implementation rather than explicit gradient-based optimization.

### 3.3. Overview of the PhyGeo-KG Framework

**Figure 1:**
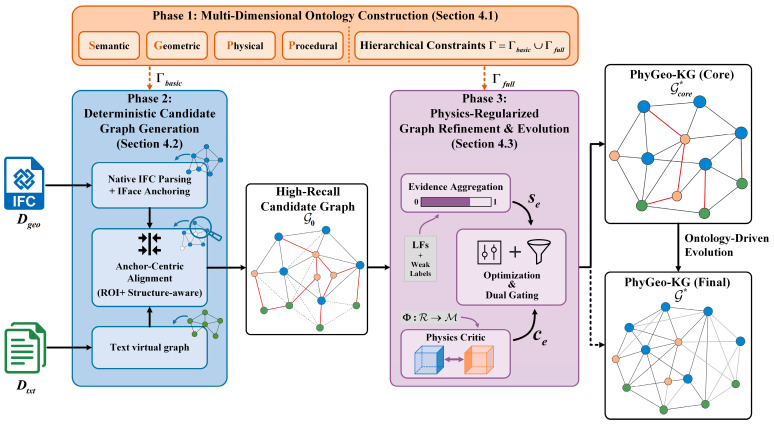
The overall framework of PhyGeo-KG construction.

To meet these objectives, this work proposes a progressive three-phase methodology for high-fidelity PhyGeo-KG construction. As shown in Figure 1, this hierarchical framework is designed to progressively filter semantic noise, bridge heterogeneous modal gaps, and inject domain-specific physical constraints, ultimately realizing interpretable and physics-compliant knowledge graph generation. The three core phases are elaborated as follows:

1. Multi-Dimensional Ontology Construction (Section 4.1): We establish a unified Semantic–Geometric–Physical–Procedural (S-G-P-P) multi-dimensional ontology representation, which serves as the theoretical cornerstone of the entire methodology. This phase introduces computable mechanism models (encoding physical interaction rules) and geometric interfaces (IFaces) (standardizing entity spatial anchoring) as executable “modal bridges.” These components effectively resolve the heterogeneity gap between unstructured text, structured geometry, and implicit physical laws, laying a computable foundation for cross-modal alignment.
2. Deterministic Candidate Graph Generation (Section 4.2): Based on the multi-dimensional ontology, we instantiate a high-recall candidate graph $G_{0}$ through structure-aware deterministic cross-modal alignment. By leveraging native IFC parsing (for geometric entity extraction) and IFace-based anchoring (for semantic–geometric mapping), this phase achieves transparent and interpretable grounding of text mentions to physical entities. Notably, this deterministic design specifically addresses the zero-shot cold-start challenge in domain-specific knowledge graph construction, ensuring high recall without relying on large-scale labeled data.
3. Physics-Regularized Graph Refinement and Evolution (Section 4.3): To prune semantic hallucinations and enhance physical consistency, we implement distant supervision with a dual semantic–geometric gating mechanism. This phase integrates the proposed Physics Critic module to perform adaptive edge pruning, followed by an ontology-driven evolution strategy to infer missing knowledge. Through this “prune-and-evolve” cycle, the initial candidate graph $G_{0}$ is refined into the final high-fidelity PhyGeo-KG $G^{*}$, balancing precision, physical compliance, and coverage.

As a mathematical foundation for the detailed methodology presented in the subsequent sections, the key notations and symbols used throughout this framework are summarized in Table 1.

## 4. Methodology: PhyGeo-KG Construction

This chapter describes the construction methodology of the PhyGeo-KG framework, structured progressively from Multi-Dimensional Ontology Development (Section 4.1) to Deterministic Candidate Graph Construction (Section 4.2) and Physics-Regularized Refinement and Evolution (Section 4.3).

### 4.1. Multi-Dimensional Ontology Construction

#### 4.1.1. Architecture Design: Integrating FBS and PPR

**Figure 2:**
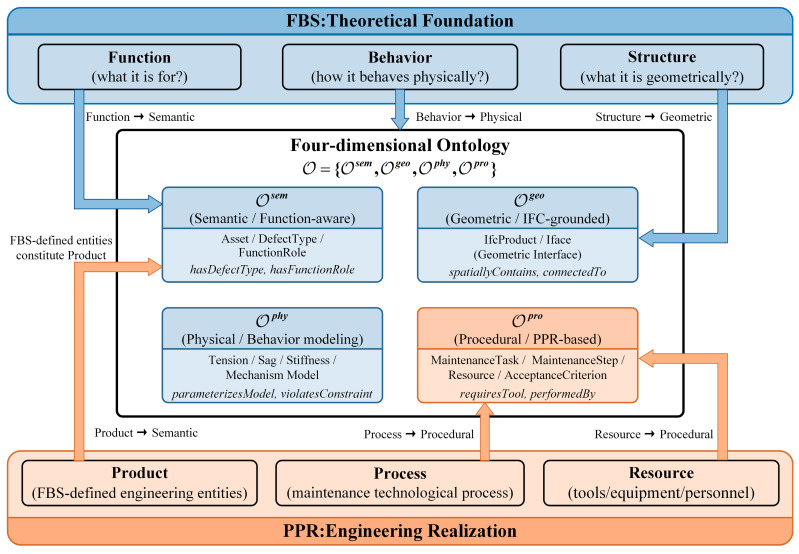
Four-dimensional ontology architecture.

Catenary maintenance involves tightly coupled attributes that cannot be expressed by a static taxonomic ontology alone. To address this, we construct a four-dimensional ontology O by integrating the Function–Behavior–Structure (FBS) [19,20] theoretical methodology with the Product–Process–Resource (PPR) [21,22] engineering paradigm. The architecture of this integration is illustrated in Figure 2.

This hybrid architecture follows a “theory-to-engineering” progression:

1. Theoretical Foundation (FBS): We use the FBS methodology to decompose the intrinsic properties of engineering entities. Structure maps to Geometry ($O^{\mathrm{geo}}$), Behavior maps to Physics ($O^{\mathrm{phy}}$), and Function maps to Semantics ($O^{\mathrm{sem}}$). This provides the ontology with a rigorous theoretical basis for describing “what the entity is.”
2. Engineering Realization (PPR): To extend the ontology into the railway maintenance domain, we overlay the PPR paradigm. The FBS-defined entities constitute the Product, while maintenance workflows and necessary tools are modeled as Process and Resource within $O^{\mathrm{pro}}$.

By fusing these two paradigms, we establish a Semantic–Geometric–Physical–Procedural (S–G–P–P) four-dimensional ontology: $O=\{O^{\mathrm{sem}},O^{\mathrm{geo}},O^{\mathrm{phy}},O^{\mathrm{pro}}\}$. This unified framework supports heterogeneous feature characterization for general engineering domains while providing specific executable interfaces for catenary maintenance.

#### 4.1.2. Sub-Ontologies with Computable Hooks

To make the multi-dimensional architecture operational, each sub-ontology is equipped with specific classes, relations, and white-box “computable hooks” that enable algorithmic processing. A comprehensive overview of these components is summarized in Table 2, with detailed formulations provided as follows:

1. Semantic Ontology $O^{\mathrm{sem}}$ (Function-aware Taxonomy). $O^{\mathrm{sem}}$ establishes a standardized taxonomy for the catenary domain, defining controlled vocabularies for (i) assets (e.g., *Dropper*, *SteadyArm*), (ii) defect types (e.g., *FatigueFracture*, *ElectricalBurn*), and (iii) functional roles (e.g., *supporting*, *positioning*, *current-carrying*). Relations such as *hasFunctionRole* and *hasDefectType* serve as semantic anchors, enabling the mapping of unstructured text mentions to formal domain concepts.
2. Geometric Ontology $O^{\mathrm{geo}}$ (IFC Grounding and IFace). $O^{\mathrm{geo}}$ formalizes IFC-grounded geometry with explicit support for computable predicates such as *spatiallyContains*, *adjacentTo*, and *connectedTo*. Crucially, it introduces the Geometric Interface (IFace) concept, which abstracts physically meaningful connection regions (e.g., *BoltHole*, *ClampSurface*, *ArticulatedJoint*) as stable structural features. Formally, an IFace is modeled as an explicit geometric entity associated with an IFC instance, serving as the minimal interaction unit for deterministic alignment and constraint evaluation.
3. Physical Ontology $O^{\mathrm{phy}}$ (Behavior Modeling and Operatorization). $O^{\mathrm{phy}}$ models physical quantities (e.g., *Tension*, *Stiffness*), boundary conditions, and governing mechanisms. Instead of merely storing scalar parameters, we define a Mechanism Model as a callable operator template that maps a geometry-defined domain and physical parameters to a physically consistent state (or residual). Concretely, for a mechanism model M, the relationships are defined in Equation (2):
  (2) u=M(Ω,p,b),  r=R(u;Ω,p,b)
  where Ω is the geometric domain, p denotes physical parameters, and b denotes boundary conditions. The output u represents the physical state field (e.g., tension distribution or displacement), and r represents the physical residual (e.g., the degree of equilibrium violation). In Section 4.3, the magnitude of r is instantiated as the physics violation $v_{\mathrm{phy}}(e)$, enabling the system to computationally verify physical consistency.
4. Procedural Ontology $O^{\mathrm{pro}}$ (PPR-based Process–Resource Modeling). $O^{\mathrm{pro}}$ formalizes maintenance technological processes as structured sequences of engineering operations. Rather than generic workflows, it encodes the rigorous steps, preconditions, and required resources defined in technical regulations. It defines key classes such as *MaintenanceTask*, *MaintenanceStep*, *Resource* (e.g., *TorqueWrench*), and *AcceptanceCriterion*. This sub-ontology enables procedural compliance reasoning, ensuring that extracted knowledge and maintenance relations align with standard operating protocols and engineering safety requirements.

#### 4.1.3. Cross-Dimensional Relations and Hierarchical Constraint Set

**Figure 3:**
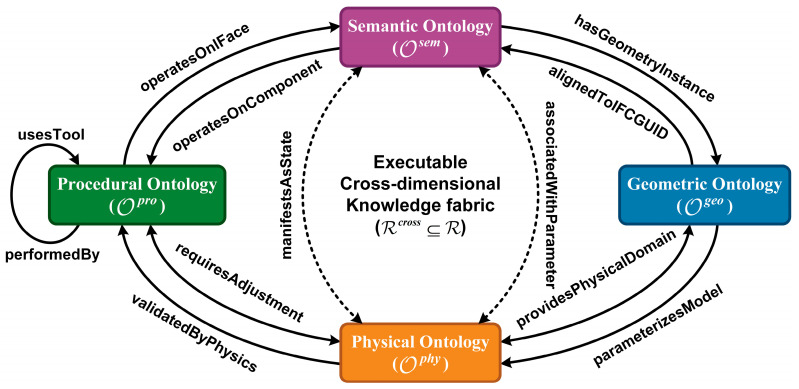
The cross-dimensional knowledge fabric.

Following the ontology-engineering notion of cross-tree relations (i.e., linking concepts across taxonomic branches) [10], we define a set of cross-dimensional relations $R^{\mathrm{cross}}\subseteq R$ to weave the four sub-ontologies into an executable knowledge fabric. As illustrated in Figure 3, these relations include:

- Semantic–Geometric: *hasGeometryInstance*, *alignedToIFCGUID* (basis for grounding in Section 4.2).
- Geometric–Physical: *providesPhysicalDomain*, *parameterizesModel* (basis for physics critic in Section 4.3).
- Semantic–Physical: *manifestsAsState*, *associatedWithParameter* (linking semantic defects to physical anomalies).
- Physical–Procedural: *validatedByPhysics*, *requiresAdjustment* (physics-driven maintenance decisions).
- Procedural–Geometry: *operatesOnComponent*, *operatesOnIFace* (action localization for refinement).
- Procedural–Resource: *usesTool*, *performedBy* (resource allocation).

These cross-dimensional relations, together with the unified multi-dimensional ontology O, provide the structural backbone for enforcing consistency across semantics, geometry, physics, and procedures. Based on O and $R^{\mathrm{cross}}$, we therefore construct a hierarchical constraint set $\Gamma =\Gamma_{\mathrm{basic}}\cup \Gamma_{\mathrm{full}}$ to explicitly separate the usage boundary between the candidate generation phase (Section 4.2) and the refinement phase (Section 4.3), as detailed in Table 3:

(i) Hard Constraints ($\Gamma_{\mathrm{basic}}$): Boolean predicates used for legality pruning in candidate graph construction (e.g., domain/range validity, IFC containment/aggregation legality, basic topological feasibility).
(ii) Soft Constraints ($\Gamma_{\mathrm{full}}$): Differentiable violation functions used for regularization and refinement (e.g., residual penalties from mechanism models, tolerance-aware inequality constraints, procedural logic constraints).

To ensure logical correctness, we checked the consistency of the constructed ontology O using the Pellet reasoner, confirming no satisfiability errors.

### 4.2. Deterministic Candidate Graph Generation

This section tackles the problem of instantiating a high-recall candidate graph $G_{0}$ from unstructured texts $D_{\mathrm{txt}}$ and IFC-compliant BIM models $D_{\mathrm{geo}}$.

The zero-shot engineering challenge in catenary maintenance is that labeled training data for cross-modal alignment is often unavailable. Consequently, end-to-end neural matching models are prone to overfitting and offer limited interpretability.

**Figure 4:**
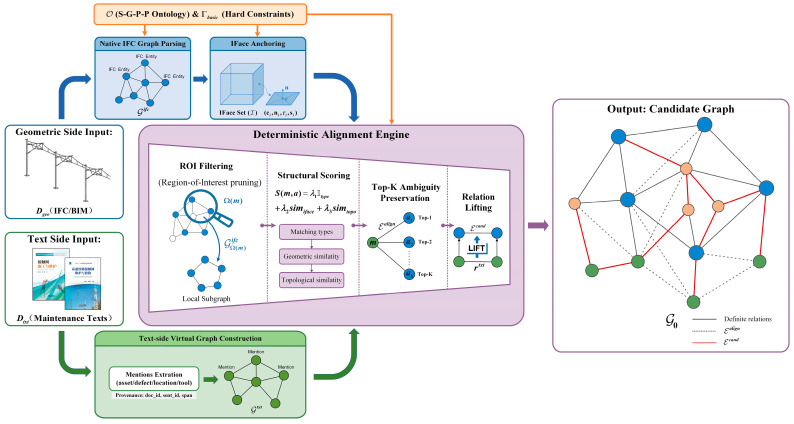
The deterministic candidate graph generation pipeline. The non-English text in the figure displays the covers of two Chinese technical manuals used as data sources. The left cover translates to Overhead Contact System Construction and Maintenance, and the right cover translates to High-Speed Railway Overhead Contact System Maintenance and Repair.

The proposed white-box strategy overcomes this limitation by introducing an anchor-centric deterministic alignment approach, whose overall pipeline is illustrated in Figure 4. Rather than relying on latent vector spaces, we parse the BIM model into a schema-faithful native graph and introduce Geometric Interfaces (IFace) as explicit anchors. This allows us to deterministically ground text mentions to physical entities based on rigorous topological and geometric matching. During this stage, we enforce only the hard constraint set $\Gamma_{\mathrm{basic}}$ for legality pruning (e.g., domain validity and basic topological feasibility), while deferring the soft constraint set $\Gamma_{\mathrm{full}}$ to Section 4.3 for physics-regularized refinement.

#### 4.2.1. Native IFC Graph Construction and IFace Anchoring

Unlike methods that flatten BIM data into sequences, we retain the rich topological structure of the Industry Foundation Classes (IFC). We parse $D_{\mathrm{geo}}$ into a native instance graph $G^{ifc}=(V^{ifc},E^{ifc})$, where the nodes ($V^{ifc}$) correspond to IFC entities (e.g., *IfcProduct*, *IfcBuildingElementProxy*) and the edges ($E^{ifc}$) correspond to explicit schema relations (e.g., *IfcRelAggregates*, *IfcRelConnects*). This representation supports the local topological reasoning needed for disambiguation.

To enable stable cross-modal grounding, we introduce IFace (defined in Section 4.1) as the minimal interaction unit. Let I denote the set of IFace nodes. Each i∈I is associated with a parent IFC instance $v\in V^{ifc}$ via (v,hasIFace,i), and is parameterized as a tuple $\mathrm{IFace}(i)=(c_{i},n_{i},\tau_{i},s_{i})$, where $c_{i}$ is the geometric centroid, $n_{i}$ is the orientation normal, $\tau_{i}$ is the interface type (e.g., *ClampSurface*, *BoltHole*), and $s_{i}$ stores scale parameters.

**Figure 5:**
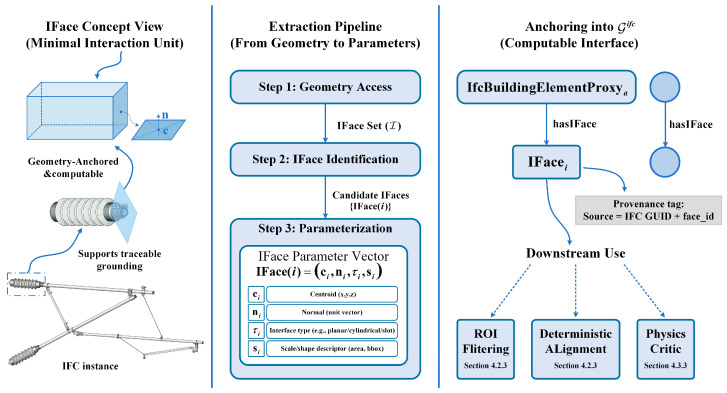
The IFace extraction and anchoring pipeline.

This concept is inspired by local attributed adjacency in CAD. By explicitly extracting these features from the IFC B-Rep (Boundary Representation) geometry, we map abstract 3D shapes to discrete, semantically meaningful anchors that can be matched against text descriptions (e.g., “loose clamp bolt”), as visualized in Figure 5.

#### 4.2.2. Text-Side Virtual Graph Construction

Before constructing the text-side virtual graph $G^{txt}$, the unstructured corpus undergoes a rigorous Natural Language Processing (NLP) pipeline. This pipeline includes document cleaning, sentence segmentation, domain-specific Named Entity Recognition (NER) guided by the $O^{sem}$ lexicon to identify specialized components, and syntactic dependency parsing (e.g., via spaCy) to extract Subject–Verb–Object (SVO) triples.

To align with the structured $G^{ifc}$, we convert unstructured maintenance logs $D_{\mathrm{txt}}$ into a text-side virtual graph $G^{\mathrm{txt}}=(V^{\mathrm{txt}},E^{\mathrm{txt}})$, where the nodes ($V^{\mathrm{txt}}$) represent extracted mentions (e.g., assets, defects, tools) typed by the semantic ontology $O^{\mathrm{sem}}$, and the edges ($E^{\mathrm{txt}}$) encode mention-level relations derived from syntactic dependency patterns (e.g., Subject–Verb–Object triples). Each node also stores provenance metadata (document ID, sentence span), ensuring that any relation extracted is traceable to its source. The detailed schema of these graph elements, along with the specific alignment constraints enforced by $\Gamma_{\mathrm{basic}}$, is summarized in Table 4.

#### 4.2.3. Deterministic Alignment and Candidate Instantiation

We instantiate the candidate graph $G_{0}$ by linking $G^{\mathrm{txt}}$ and $G^{ifc}$ via a three-step deterministic procedure.

1. Spatially constrained pruning (region of interest): To reduce the search space and limit spatial ambiguity, we parse location cues (e.g., “Span 135”, “Pole 22”) from the text to define a region of interest (ROI), Ω(m). We then extract a local subgraph $G_{\Omega (m)}^{ifc}\subset G^{ifc}$. This filters out geometrically similar yet spatially irrelevant instances (e.g., identical droppers on different spans).
2. Subgraph-aware structural scoring: Within the ROI, we rank candidate anchors $a\in G_{\Omega (m)}^{ifc}$ using a white-box scoring function S(m,a) that combines type, interface, and structural similarity, as defined in Equation (3):
  (3) $$S(m,a)=\lambda_{1}\cdot I_{\mathrm{type}}(m,a)+\lambda_{2}\cdot {\mathrm{sim}}_{\mathrm{iface}}(m,a)+\lambda_{3}\cdot {\mathrm{sim}}_{\mathrm{topo}}(N_{k}(m),N_{k}(a))$$
  where $\lambda_{1},\lambda_{2},\lambda_{3}$ are hyperparameters controlling the weight of each factor; $I_{\mathrm{type}}(m,a)$ is an indicator function that returns 1 if the ontology type of mention m matches the IFC type of anchor a and 0 otherwise; ${\mathrm{sim}}_{\mathrm{iface}}$ is the Jaccard similarity between the interface types implied by the text (e.g., “clamp”) and the IFaces attached to the candidate geometry; and ${\mathrm{sim}}_{\mathrm{topo}}$ is the cosine similarity of topological signature vectors derived from the k-hop neighborhoods $N_{k}(m)$ and $N_{k}(a)$. Mathematically, the interface similarity ${\mathrm{sim}}_{\mathrm{iface}}$ is computed using the Jaccard index between the text-implied interface set $T_{m}$ and the geometric interface set $T_{a}$, as formulated in Equation (4):
  (4) $${\mathrm{sim}}_{\mathrm{iface}}(m,a)=\frac{\left|T_{m}\cap T_{a}\right|}{\left|T_{m}\cup T_{a}\right|}$$

The topological similarity ${\mathrm{sim}}_{\mathrm{topo}}$ is computed using the Cosine similarity of the structural signature vectors $z_{m}$ and $z_{a}$ derived from their k-hop neighborhoods, as expressed in Equation (5):

(5) $${\mathrm{sim}}_{\mathrm{topo}}(N_{k}(m),N_{k}(a))=\frac{z_{m}^{⊤}z_{a}}{\|z_{m}{\|}_{2}\|z_{a}{\|}_{2}}$$

3. Candidate lifting and instantiation: We retain the Top-K ranked anchors to create alignment links $E^{align}$. Finally, we project the text-level relations onto the aligned physical anchors to generate the candidate semantic relations $E^{\mathrm{cand}}$, as shown in Equation (6):

(6) $$E^{\mathrm{cand}}=\{(a_{u},r,a_{v})\;|\;(m_{u},r^{\mathrm{txt}},m_{v})\in E^{\mathrm{txt}},a_{u}\in \mathrm{TopK}(m_{u}),a_{v}\in \mathrm{TopK}(m_{v})\}$$

where TopK(⋅) returns the set of highest-scoring anchor candidates. This procedure yields a high-recall candidate graph $G_{0}$, which serves as the input for the physics-regularized refinement in Section 4.3.

### 4.3. Physics-Regularized Graph Refinement and Evolution

This section describes the progressive transformation of the high-recall candidate graph $G_{0}$ into the refined core $G_{\mathrm{core}}^{*}$ and ultimately the final evolved graph $G^{*}$.

A central limitation of traditional distant supervision is its reliance on deep neural models, such as CNNs [35] and PCNNs [36], to encode semantic features. However, in safety-critical domains with severe label sparsity, such as catenary maintenance engineering, these opaque models can overfit and lack the transparency required for engineering validation.

**Figure 6:**
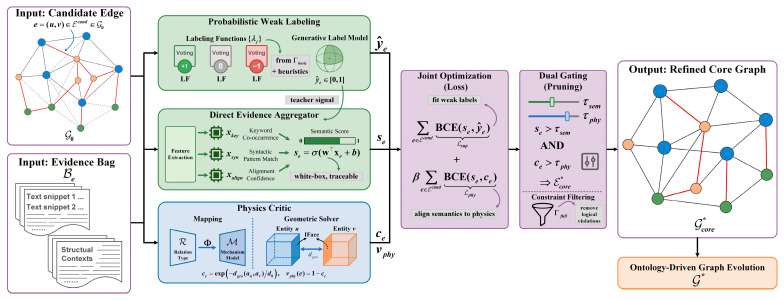
The physics-regularized graph refinement and evolution pipeline.

The white-box instantiation addresses this limitation by proposing a physics-regularized refinement and evolution strategy, whose overall pipeline is illustrated in Figure 6. Opaque neural encoders are replaced with a direct evidence aggregator, and physical regularization is instantiated as an explicit geometric constraint solver. This ensures that every edge in the refined core $G_{\mathrm{core}}^{*}$ is supported by traceable semantic evidence and validated by rigorous physical logic, providing a trustworthy foundation for the subsequent ontology-driven expansion.

#### 4.3.1. Evidence Aggregation and Probabilistic Weak Labeling

Because manual annotation is infeasible at scale, we adopt distant supervision. For each candidate edge $e\in E^{\mathrm{cand}}$, an edge-level evidence bag $B_{e}$ is constructed, which collects all retrieved text snippets and structural contexts associated with the entity pair.

To generate initial supervision signals, a set of labeling functions (LFs) ${\left\{\lambda_{j}\right\}}_{j=1}^{J}$ is defined, inspired by the Hard Constraint Set $\Gamma_{\mathrm{basic}}$ and domain heuristics, as summarized in Table 5. Each LF outputs a discrete vote {−1,0,1}. These noisy votes are aggregated using a generative label model to obtain a probabilistic weak label ${\hat{y}}_{e}\in [0,1]$. Here, ${\hat{y}}_{e}$ represents the estimated probability that relation e is true based solely on heuristic rules, serving as the “teacher” signal for the semantic aggregator.

The labeling functions (LFs) were designed from three complementary sources: ontology/schema constraints, geometry/IFace-based physical cues, and domain-specific linguistic patterns extracted from maintenance documents. While Table 5 presents representative examples, our implementation utilizes a total of 15 distinct LFs. The overall empirical coverage of these LFs (the proportion of candidate edges triggered by at least one LF) is approximately 42%. To handle incorrect or conflicting LFs, our system utilizes a generative weak-supervision layer rather than hard majority voting. This generative model probabilistically aggregates conflicting LF outputs, thereby reducing the influence of noisy or inconsistent LF signals. This probabilistic aggregation intrinsically improves the system’s robustness to suboptimal rules, though exhaustive manual LF-group ablations remain a valuable direction for future validation. Furthermore, to ensure weak-label reliability, we conducted a manual audit on a sampled 10% subset of candidate edges, confirming high agreement between the probabilistically aggregated weak labels and expert judgment.

#### 4.3.2. The Semantic Encoder: Direct Evidence Aggregator

To compute the semantic confidence score $s_{e}=f_{\theta }(e)$, opaque latent embeddings are avoided in favor of an interpretable feature-vector construction. For each candidate edge e=(u,v), we construct a feature vector $x_{e}\in {\mathbb{R}}^{d}$ derived explicitly from the evidence bag $B_{e}$. The key dimensions include (i) $x_{key}$ (keyword co-occurrence), which is the normalized frequency of domain-specific interaction keywords (e.g., “fracture”, “loosen”, defined in $O^{\mathrm{sem}}$) appearing in the context window between mentions of u and v; (ii) $x_{syn}$ (syntactic pattern match), which is a binary indicator (1 or 0) of whether the dependency path between u and v matches predefined linguistic templates (e.g., Subject[*Asset_A*]–Verb[*Connects*]–Object[*Asset_B*]); and (iii) $x_{align}$ (alignment confidence), which is the structural alignment score derived in Section 4.2.3, defined as $x_{align}=\min(S(m_{u},a_{u}),S(m_{v},a_{v}))$, representing the grounding reliability of the connected entities.

The semantic confidence $s_{e}$ is then computed via a linear aggregator calibrated by a sigmoid function, according to Equation (7):

(7) $$s_{e}=\sigma (w^{⊤}x_{e}+b)$$

where $w\in {\mathbb{R}}^{d}$ and b∈ℝ denote explicit aggregation weights and a bias term calibrated against the weak labels, and σ(⋅) is the sigmoid function mapping the output to (0,1). This direct evidence aggregator ensures that the semantic score $s_{e}$ is linearly decomposable and directly traceable to specific linguistic or structural features.

#### 4.3.3. The Physics Critic: Mechanism-Based Regularization

A key innovation of PhyGeo-KG is the physics critic, an operator that evaluates the physical plausibility of a candidate relation and imposes the penalty term $L_{\mathrm{phy}}$. We first define a mapping Φ:R→M that links each semantic relation type r (e.g., mechanically *ConnectedTo*) to a governing mechanism model (e.g., geometric connectivity model), as defined in Section 4.1.2.

Although the general framework can support PDE-based residuals, the primary constraint for static catenary maintenance is topological integrity. Accordingly, we instantiate the physics critic as an explicit geometric constraint solver. For a candidate edge e=(u,v) linked to geometric anchors $a_{u}$ and $a_{v}$, the solver computes a physical plausibility score $c_{e}\in (0,1]$ based on the characteristic interaction scale ($d_{0}$), which is calculated using Equation (8):

(8) $$c_{e}=\exp\left(-\frac{d_{\mathrm{geo}}(a_{u},a_{v})}{d_{0}}\right),\;v_{\mathrm{phy}}(e)=1-c_{e}$$

where $d_{\mathrm{geo}}(a_{u},a_{v})$ is the Euclidean distance between the centroids of the IFaces associated with anchors $a_{u}$ and $a_{v}$, and $v_{\mathrm{phy}}(e)$ is the degree of physical violation, which serves as the regularization penalty.

This formulation behaves as a differentiable soft logic gate. If a relation is physically consistent ($d_{\mathrm{geo}}\ll d_{0}$), then $c_{e}\to 1$ and $v_{\mathrm{phy}}\to 0$, so the regularization term vanishes and the semantic score $s_{e}$ dominates. Conversely, if the relation is physically impossible ($d_{\mathrm{geo}}\gg d_{0}$), then $c_{e}\to 0$ and $v_{\mathrm{phy}}\to 1$, imposing a high penalty that forces the model to suppress $s_{e}$ regardless of semantic strength. This mechanism explicitly penalizes the long-distance semantic bias (detailed in Section 5), forcing the model to distinguish genuine connections from semantic hallucinations.

#### 4.3.4. Surrogate Optimization and Dual Gating Refinement

To formalize the desired trade-off between semantic evidence and physical plausibility formulated in Section 3.2, we introduce the physics-regularized surrogate objective in Equation (9):

(9) $$\underset{w,b}{\min}\;\sum_{e\in E^{\mathrm{cand}}}\underset{L_{\sup}}{\underset{︸}{\mathrm{BCE}(s_{e},{\hat{y}}_{e})}}+\beta \sum_{e\in E^{\mathrm{cand}}}\underset{L_{\mathrm{phy}}}{\underset{︸}{\mathrm{BCE}(s_{e},c_{e})}}$$

where BCE denotes the binary cross-entropy loss. The first term $L_{\sup}$ encourages the model to fit the heuristic weak labels (${\hat{y}}_{e}$), while the second term $L_{\mathrm{phy}}$ (weighted by hyperparameter β) regularizes the semantic confidence $s_{e}$ to align with the physical plausibility $c_{e}$.

Theoretically, the choice of the hyperparameter β plays a critical role in determining the final content of the core graph by controlling the strictness of physical governance. A larger β forces the objective to heavily penalize relations with high physical residuals, resulting in a sparser but highly precise and physically compliant core graph. Conversely, a smaller β diminishes the influence of the physics critic; while this may increase relation coverage, it inevitably re-introduces long-distance semantic hallucinations. Equation (9) should therefore be interpreted as the general regularized formulation of the PhyGeo-KG scoring optimization. In the current study, we adopt a deterministic white-box engineering instantiation to approximate this optimization problem, rather than using explicit gradient-based solvers.

After calibrating the scorer, we approximate the discrete edge-selection objective (Equation (1)) to obtain the refined core graph $G_{\mathrm{core}}^{*}$. We employ a dual semantic–geometric gating mechanism, retaining an edge if and only if it satisfies both the semantic and physical thresholds, as formulated in Equation (10):

(10) $$E_{\mathrm{core}}^{*}=\{e\in E^{\mathrm{cand}}\;|s_{e}>\tau_{\mathrm{sem}}\;\mathrm{AND}\;c_{e}>\tau_{\mathrm{phy}}\}$$

where $\tau_{\mathrm{sem}}$ and $\tau_{\mathrm{phy}}$ are user-defined acceptance thresholds. Finally, to satisfy the logical condition (s.t. E satisfies Γ) defined in Equation (1), we apply post hoc constraint filtering using the hierarchical constraint set Γ (e.g., cardinality restrictions, procedural logic). This ensures the final refined core graph is semantically supported, geometrically bounded, and logically compliant. The complete procedure, from deterministic candidate generation to the final physics-regularized refinement, is summarized in Algorithm 1.
**Algorithm 1** Deterministic Candidate Generation and Physics-Regularized Refinement$\mathrm{Input}:\;\mathrm{Maintenance}\;\mathrm{records}\;D_{\mathrm{txt}}$$,\;\mathrm{BIM}/\mathrm{IFC}\;\mathrm{models}\;D_{\mathrm{geo}}$, multi-dimensional ontology O$,\;\mathrm{ontology}\;\mathrm{constraints}\;\Gamma =\{\Gamma_{\mathrm{basic}},\Gamma_{\mathrm{full}}\}$$,\;\mathrm{gating}\;\mathrm{thresholds}\;\tau_{\mathrm{sem}},\tau_{\mathrm{phy}}$$\mathrm{Output}:\;\mathrm{Refined}\;\mathrm{core}\;\mathrm{graph}\;G_{\mathrm{core}}^{*}$1: $G^{ifc}\leftarrow \;\mathrm{Parse}\_\mathrm{IFC}\;(D_{\mathrm{geo}})$                        ⊳ Extract native IFC topology and B-Rep2: $G^{\mathrm{txt}}\leftarrow \;\mathrm{NLP}\_\mathrm{Pipeline}\;(D_{\mathrm{txt}})$                       ⊳ Extract mentions and raw semantic triples3: $\mathrm{Initialize}\;\mathrm{candidate}\;\mathrm{edge}\;\mathrm{set}\;E^{\mathrm{cand}}\leftarrow \emptyset$4: $\mathrm{for}\;\mathrm{each}\;\mathrm{triple}\;(m_{u},r,m_{v})\in E^{\mathrm{txt}}$ **do**5:  **if** relation type r is incompatible with ontology priors in O **then**6:   **continue**7:  **end if**8:  $\mathrm{Induce}\;\mathrm{spatial}\;\mathrm{Regions}\;\mathrm{of}\;\mathrm{Interest}\;(\mathrm{ROI})\;\Omega (m_{u})$$\;\mathrm{and}\;\Omega (m_{v})$ from maintenance context ⊳  Spatial pruning9:  $\mathrm{Align}\;m_{u},m_{v}$$\;\mathrm{to}\;\mathrm{anchor}-\mathrm{bearing}\;\mathrm{IFC}\;\mathrm{instances}\;a_{u},a_{v}\in V^{ifc}$ via IFace-aware scoring (Equation (3))10:  $\mathrm{Formulate}\;\mathrm{grounded}\;\mathrm{candidate}\;\mathrm{edge}\;e=(a_{u},r,a_{v})$11:  **if** e$\;\mathrm{satisfies}\;\mathrm{basic}\;\mathrm{ontology}\;\mathrm{constraints}\;\Gamma_{\mathrm{basic}}$ **then** 12:  $E^{\mathrm{cand}}\leftarrow E^{\mathrm{cand}}\cup \{e\}$ (Equation (6))                             ⊳ Hard legality filtering13:  **end if**14: **end for**15: $\mathrm{Derive}\;\mathrm{weak}\;\mathrm{labels}\;{\hat{y}}_{e}$ using J domain-specific labeling functions (LFs)              ⊳ Distant supervision16: $\mathrm{for}\;\mathrm{each}\;\mathrm{candidate}\;\mathrm{edge}\;e\in E^{\mathrm{cand}}$
**do**17:  $\mathrm{Aggregate}\;\mathrm{explicit}\;\mathrm{evidence}\;\mathrm{to}\;\mathrm{compute}\;\mathrm{semantic}\;\mathrm{confidence}\;s_{e}$ (Equation (7))18:  $\mathrm{Invoke}\;\mathrm{the}\;\mathrm{Physics}\;\mathrm{Critic}\;\mathrm{to}\;\mathrm{compute}\;\mathrm{physical}\;\mathrm{plausibility}\;c_{e}$ (Equation (8))             ⊳ Mechanism regularization19:  $\mathrm{Compute}\;a\;\mathrm{regularized}\;\mathrm{confidence}\;\mathrm{signal}\;\mathrm{by}\;\mathrm{coupling}\;{\hat{y}}_{e}$$,\;s_{e}$$,\;\mathrm{and}\;c_{e}$ (Equation (9))         ⊳White-box regularization20: **end for**21: $E_{\mathrm{core}}^{*}\leftarrow \{e\in E^{\mathrm{cand}}|s_{e}\geq \tau_{\mathrm{sem}}\;\mathrm{AND}\;c_{e}\geq \tau_{\mathrm{phy}}\}$ (Equation (10))                    ⊳ Dual semantic–geometric gating22: $\mathrm{Apply}\;\Gamma_{\mathrm{full}}$$\;\mathrm{to}\;\mathrm{perform}\;\mathrm{conflict}\;\mathrm{resolution}\;\mathrm{and}\;\mathrm{de}-\mathrm{duplication}\;\mathrm{on}\;E_{\mathrm{core}}^{*}$            ⊳ Post hoc logic constraints23: $\mathrm{return}\;G_{\mathrm{core}}^{*}=(V^{ifc},E_{\mathrm{core}}^{*})$

#### 4.3.5. Ontology-Driven Graph Evolution

To mitigate the sparsity of $G_{\mathrm{core}}^{*}$, we define an ontology-driven evolution mechanism that expands the graph scope without introducing noise, resulting in the final graph $G^{*}$. We first utilize the structural patterns encoded in the multi-dimensional ontology O as inference templates. Let $T=(C_{u},r,C_{v})$ be a valid triple pattern in O, where $C_{u},C_{v}\in V_{O}$ denotes ontology classes (e.g., *Dropper*, *ContactWire*) and r is a relation type. If the refined graph contains a path that logically implies this pattern (e.g., via inverse or transitive properties defined in $O^{\mathrm{pro}}$ or $O^{\mathrm{geo}}$), we instantiate a missing edge candidate $e_{\mathrm{new}}$.

Furthermore, PhyGeo-KG implements a mandatory dual-safeguard mechanism to prevent error propagation during chain inference. Logically, newly inferred relations must strictly follow the predefined inference templates encoded within the S-G-P-P ontology, ensuring schema compliance. Physically, any newly inferred edge $e_{\mathrm{new}}$ must still pass the exact same physics critic check. Even if a relation is logically implied by the ontology, it is admitted to the final graph only if $v_{\mathrm{phy}}(e_{\mathrm{new}})<1-\tau_{\mathrm{phy}}$. This ensures that the evolution of the graph remains physically bounded and does not propagate logical errors into physically impossible regions. This cycle enables PhyGeo-KG to grow from a sparse set of high-confidence seeds into a dense, connected knowledge network.

## 5. Experiments and Case Study

This chapter systematically validates the proposed PhyGeo-KG framework using a real-world catenary maintenance dataset. The experimental design follows a progressive path from noise filtering to mechanism consistency, and finally to knowledge evolution. Specifically, this chapter addresses the following three core research questions (RQs):

1. RQ1 (Effectiveness verification): Compared to traditional pure text extraction methods, can the distant supervision mechanism with physical regularization effectively suppress semantic noise and improve the precision and physical consistency of relation extraction?
2. RQ2 (Mechanism analysis): What mechanism do the physics critic and its penalty term ($v_{\mathrm{phy}}$) play in distinguishing valid physical connections from semantic hallucinations?
3. RQ3 (Evolution and application): Based on the ontology-driven evolution mechanism proposed in Section 4.3.5, can the framework discover latent structural patterns and achieve effective graph scale growth while maintaining physical compliance?

### 5.1. Experimental Setup

#### 5.1.1. Dataset Construction: Catenary-KG-2K

To evaluate engineering applicability, this paper constructs a domain-specific dataset, Catenary-KG-2K, containing multi-source heterogeneous data. The dataset covers the design and maintenance data of a 2 km section of a high-speed railway. Specifically, the geometric data ($D_{\mathrm{geo}}$) were parsed from high-precision BIM models adhering to the IFC standard, ensuring 100% IFace generation coverage; the text data ($D_{\mathrm{txt}}$) were sourced from 350 real-world maintenance documents, including construction logs, maintenance regulations, and technical manuals of the line. Detailed statistics of the dataset are summarized in Table 6.

#### 5.1.2. Implementation Details

Following the general framework and the engineering instantiation design philosophy established in Section 3.1, and considering the label sparsity of catenary maintenance data as well as the static and rigid nature of the system’s topology, the experiment adopts a white-box engineering instantiation strategy to implement the PhyGeo-KG framework. This strategy replaces black-box neural networks with interpretable algorithms, configured as follows:

- Semantic encoder: instantiated as the direct evidence aggregator proposed in Section 4.3.2. Addressing the cold-start challenge, we construct feature vectors by aggregating domain keyword co-occurrence frequencies ($x_{key}$) and syntactic patterns ($x_{syn}$), and use logistic regression to generate traceable semantic scores $s_{\mathrm{sem}}$
- Physics critic: instantiated as the explicit geometric constraint solver defined in Section 4.3.3. Given the static structural characteristics of the catenary system, we model physical constraints as soft logic gates based on the characteristic interaction scale ($d_{0}$). For a candidate edge e, the physical consistency score $c_{e}$ and physical violation $v_{\mathrm{phy}}$ are calculated following Equation (8). In this experiment, the characteristic interaction scale is set to $d_{0}=20.0$ m. This threshold is derived from the empirical half-wavelength of a standard catenary span (typically 40–50 m, according to the design code TB 10,009 [37]), aiming to serve as a soft threshold to effectively distinguish strong intra-span associations from weak inter-span associations.
- Hyperparameter Settings: In alignment with our white-box proxy implementation, gradient-based hyperparameters are not applicable. Instead, the framework relies on explicit engineering hyperparameters. For deterministic grounding, the structural scoring weights were empirically set to $\lambda_{1}=0.5,\;\lambda_{2}=0.3,\;\lambda_{3}=0.2$ based on validation set tuning, with a topological neighborhood depth k=2 (2-hop), and the alignment score threshold was set to 0.75. During the physics-regularized refinement, the characteristic interaction scale was set to $d_{0}=20.0$ m, a value rigorously evaluated via the sensitivity analysis in Section 5.3. The physical balancing parameter was set to β=1.0. The final dual gating thresholds for core graph inclusion were configured as $\tau_{\mathrm{sem}}=0.35$ and $\tau_{\mathrm{phy}}=0.5$. The scaling factors for physical heuristics were configured as α=2.0 and κ=0.5

#### 5.1.3. Baselines

To isolate the contribution of each module, the experiment employs a progressive ablation comparison scheme:

1. Text-only (baseline): This corresponds to the raw semantic graph $G_{raw}$. Generated solely based on semantic co-occurrence and rule templates, this represents the baseline performance without introducing geometric or physical constraints.
2. Geo-aware (baseline): This corresponds to the initial candidate graph $G_{0}$ generated in Section 4.2. This baseline introduces deterministic geometric anchoring to prune phantom nodes that cannot be grounded in the BIM model, applying only hard constraints ($\Gamma_{\mathrm{basic}}$) for pruning without soft physical regularization.
3. PhyGeo-KG (core): This corresponds to the refined core graph $G_{\mathrm{core}}^{*}$ output in Section 4.3.4. We select this intermediate high-precision kernel for comparison to ensure a fair assessment against extraction-based baselines (which lack evolution mechanisms).

#### 5.1.4. Evaluation Metrics

To evaluate graph quality across multiple dimensions, this paper adopts three core metrics:

1. Precision, Recall, and F1-score: Precision measures the proportion of correctly identified relations among the extracted candidates. For the high-confidence core graph $G_{core}^{*}$ (N=83), we report Precision@50, corresponding to a sampling rate of over 60%. Statistical analysis shows that at a 95% confidence level, the confidence interval for Precision@50 is [0.82, 0.98] (based on binomial distribution approximation, standard error ≈0.04), confirming the high statistical significance of the result. Furthermore, to quantitatively evaluate the fundamental trade-off between precision improvement and the potential loss of valid relations caused by strict physical filtering, we additionally introduce Recall and F1-score. Given the absence of complete ground truth labels for the entire massive dataset, these metrics were evaluated on the complete subset of 120 geometrically grounded candidate edges (the Geo-aware baseline). By manually annotating this subset, we established a local ground truth to rigorously track how many valid relations were retained or incorrectly pruned by the Physics Critic. The F1-score is computed as the harmonic mean of Precision and Recall to provide a comprehensive evaluation, as defined in Equation (11):
  (11) $$F1=2\times \frac{\mathrm{Precision}\times \mathrm{Recall}}{\mathrm{Precision}+\mathrm{Recall}}$$
2. Physical consistency rate (PCR): defined as the proportion of edges in the evaluated edge set $E_{\mathrm{eval}}$ that satisfy a strict geometric constraint threshold (set to $\tau_{\mathrm{geo}}=5$ m), which is calculated using Equation (12):
  (12) $$\mathrm{PCR}=\frac{\left|\left\{e\in E_{\mathrm{eval}}|\mathrm{dist}(e)\leq \tau_{\mathrm{geo}}\right\}\right|}{\left|E_{\mathrm{eval}}\right|}$$
  where $E_{\mathrm{eval}}$ is a general variable referring to the edge set of $G_{raw}$, $G_{0}$, or $G_{\mathrm{core}}^{*}$ depending on the evaluation stage. Here, $\tau_{\mathrm{geo}}=5$ m is set as a strict post hoc evaluation criterion, significantly stricter than the characteristic interaction scale ($d_{0}=20$ m) used during training, to measure whether entity pairs have achieved tight geometric adjacency.
3. Hallucination removal rate (HRR): measures the model’s ability to prune erroneous edges with high semantic scores but high physical residuals from the candidate graph $G_{0}$, as defined in Equation (13):
  (13) $$\mathrm{HRR}=\frac{\left|E_{\mathrm{hall}}\setminus E_{\mathrm{core}}^{*}\right|}{\left|E_{\mathrm{hall}}\right|}$$
  where $E_{\mathrm{hall}}=\{e\in E^{\mathrm{cand}}|s_{\mathrm{sem}}(e)\geq \tau_{\mathrm{sem}}\wedge c_{e}(e)\leq \tau_{\mathrm{phy}}\}$ is the set of identified hallucinations (see Definition 1 in Section 5.3).

### 5.2. RQ1: Effectiveness Evaluation

#### 5.2.1. Noise Filtering Mechanism Analysis

**Figure 7:**
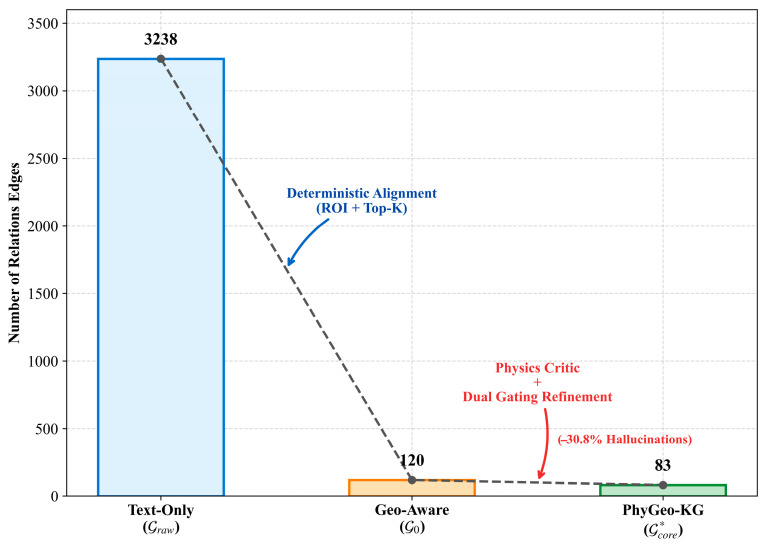
The filtering funnel of PhyGeo-KG.

The core advantage of PhyGeo-KG lies in its “funnel-like” filtering capability against massive semantic noise. This progressive filtering is particularly important for high-noise engineering datasets. As quantified in Figure 7, the construction process operates as a progressive sieve: starting from massive raw extractions, it sequentially integrates geometric anchoring and physical regularization to distill the final refined graph.

The results show that the Text-Only baseline initially generated 3238 noisy candidate edges. Deterministic anchoring acts as a crucial coarse-grained filter, reducing these edges to 120 (approximately 3.7%) by isolating only physically grounded instances. This disparity indicates that natural language text contains a large number of generalized descriptions (non-specific instances, e.g., “inspect pillars”) or mentions of components missing from the model; using text results directly would lead to a graph filled with virtual nodes that cannot be located.

This massive reduction allows the finer-grained physics critic to efficiently target the remaining candidates, ultimately distilling 83 highly reliable edges. Specifically, in this Physics Regularization stage, the differentiable physical constraints further pruned approximately 30.8% of the grounded relations. The pruned edges are typical “Semantic Hallucinations”—erroneous relations where text appears relevant (e.g., co-occurring in the same sentence) but the entities are far apart in physical space, violating the connection logic of the catenary system.

#### 5.2.2. Accuracy and Consistency Assessment

To quantitatively evaluate graph quality, we assessed metrics for the outputs of the three stages, as summarized in Table 7.

As shown in Table 7, we report Precision@50 to evaluate the absolute reliability of the top-ranked relations in the entire graph. Furthermore, the evaluation on the fully annotated subset of 120 geometrically grounded candidate edges reveals a highly beneficial progressive trend: while the strict physical regularization of PhyGeo-KG leads to a minor decrease in Recall (from 1.00 to 0.94) compared to the Geo-aware baseline, the substantial improvement in Subset Precision (from 0.67 to 0.90) allows PhyGeo-KG to achieve the highest overall F1-score (0.92).

The data show that the graph generated by the text-only method contains a large number of long-distance connections (average distance 19.651 m), with a PCR of only 34%, suggesting that most extracted relations are physically unreliable. By contrast, PhyGeo-KG achieved a significant improvement. First, precision increased from 0.56 to 0.90, proving that physical constraints effectively removed semantic false positives. Second, regarding physical compactness, the PCR increased to 86%, and the average distance was significantly compressed to 3.366 m. This suggests that relations in the final graph are highly concentrated within the effective connection range of catenary components.

Notably, the PCR is 86% rather than 100%. This result is highly consistent with the soft logic gate setting proposed in Section 4.3.3, as the algorithm does not enforce a hard cutoff but allows for the retention of a very small number of edges that are slightly farther in geometric distance (>5 m) but possess an extremely strong semantic evidence chain ($s_{\mathrm{sem}}\gg \tau_{\mathrm{sem}}$). This strategy effectively avoids erroneous exclusion (false negatives) caused by rigid geometric thresholds, demonstrating the robustness of the model under multi-modal conflicts.

### 5.3. RQ2: Mechanism Analysis

To clarify the role of the physical regularization term ($v_{\mathrm{phy}}$), we first formally define the concept of a semantic hallucination edge.

**Definition** **1.** *Semantic hallucination edge. A semantic hallucination edge refers to an erroneous candidate relation that is predicted with high confidence based solely on text co-occurrence features (* $s_{\mathrm{sem}}\geq \tau_{\mathrm{sem}}$ *) but possesses a high degree of violation in physical space (* $v_{\mathrm{phy}}\geq 1-\tau_{\mathrm{phy}}$ *, i.e.,* $c_{e}\leq \tau_{\mathrm{phy}}$ *). In this experiment, we set* $\tau_{\mathrm{sem}}=0.7$ *and* $\tau_{\mathrm{phy}}=0.5$ *, which corresponds to a geometric distance threshold of approximately 13.9 m.*

**Figure 8:**
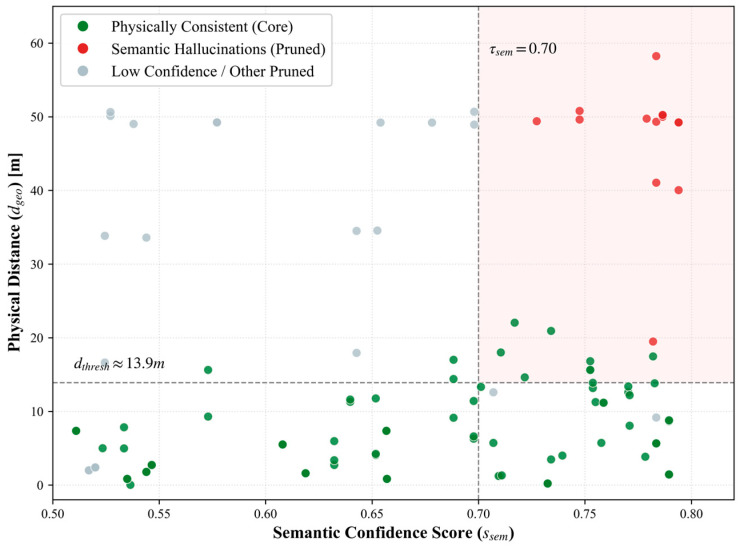
Semantic–spatial distribution and decision boundary.

We analyzed the joint distribution between semantic confidence ($s_{\mathrm{sem}}$) and physical distance ($d_{\mathrm{geo}}$) for all candidate relations. As visualized in Figure 8, we observed a significant phenomenon in the semantic–spatial distribution plot: a dense cluster of data points appears in the top-right corner, characterized by extremely high semantic scores ($s_{\mathrm{sem}}\geq 0.7$) but abnormal physical distances ($d_{\mathrm{geo}}>13.9$ m). These high-confidence noise edges typically originate from juxtaposed descriptions of equipment across different spans in maintenance logs, which traditional text models easily misjudge as strongly correlated. By introducing the physical penalty term $v_{\mathrm{phy}}$, the physics critic module successfully identified and suppressed these anomalies (marked as the red shaded region in the Figure 8), achieving an overall hallucination removal rate (HRR) of 79.4%. This quantitatively demonstrates that nearly 80% of potential semantic hallucinations—which would otherwise bypass text-only models—were successfully intercepted by physical rules before contaminating the final graph.

To verify these observations statistically, we calculated the Pearson correlation coefficient between $s_{\mathrm{sem}}$ and $d_{\mathrm{geo}}$ in the candidate edge set. The results show a statistically significant weak positive correlation between the two variables (r=0.23, p=0.013<0.05). This counterintuitive pattern reveals a significant long-distance semantic bias in pure text models—a tendency to assign higher semantic confidence to entity pairs that are spatially distant and often physically infeasible. These findings indicate that relying solely on semantic evidence cannot guarantee physical rationality and instead introduces systemic noise, thus strongly supporting the necessity of physical regularization.

Furthermore, to evaluate the sensitivity of the physical regularization and its impact on the fundamental trade-off between precision and recall, we varied the characteristic interaction scale $d_{0}$ from 10 m to 30 m, as detailed in Table 8. The metrics reveal a clear and smooth trade-off trajectory. Smaller $d_{0}$ values (e.g., 10 m) impose extremely strict physical regularization, drastically reducing the number of retained edges to 57. While this yields near-perfect Subset Precision (0.96) and Hallucination Removal Rate (HRR, 94.4%), it aggressively over-suppresses valid borderline relations, causing Subset Recall to drop significantly to 0.69. Conversely, larger values (e.g., 30 m) excessively relax the physics critic, retaining 107 edges. This allows a near-perfect Subset Recall (0.99) but fails to intercept long-distance hallucinations, resulting in poor Subset Precision (0.74) and HRR (45.2%). The configuration of $d_{0}=20$ m achieves the optimal balance, retaining 83 high-confidence edges and maximizing the overall Subset F1-score at 0.92. This empirically justifies our parameter selection based on the half-wavelength of standard catenary spans.

### 5.4. RQ3: Evolution and Application

#### 5.4.1. Ontology-Driven Graph Evolution: Scale Expansion and Physical Validation

Beyond deconstruction and refinement, PhyGeo-KG utilizes the ontology-driven evolution mechanism (proposed in Section 4.3.5) to achieve graph self-growth. Starting from the sparse refined core ($G_{\mathrm{core}}^{*}$, N=83), the system mined 24 high-confidence structural templates through template-based inference and inferred 1958 new relations via physics-consolidated expansion.

**Figure 9:**
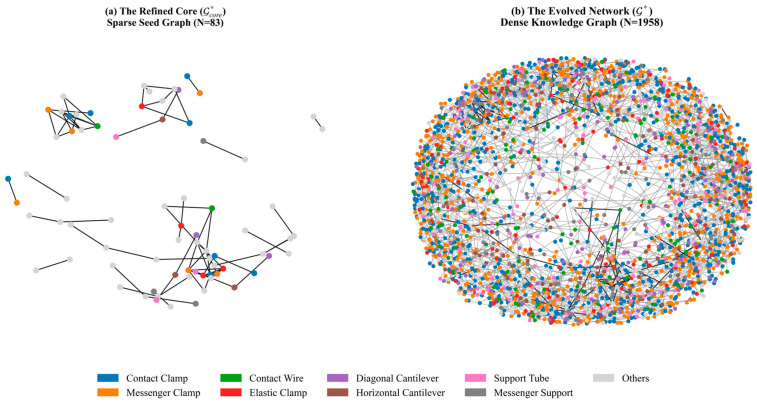
Visualization of graph topology evolution: (**a**) the refined core ($G_{\mathrm{core}}^{*}$) acting as the sparse initial seed graph; (**b**) the final evolved network ($G^{*}$).

This process resulted in a 23.6-fold growth in graph scale. As visualized in Figure 9, the graph transforms from a sparse set of high-confidence seed relations (Figure 9a) into a dense, connected semantic network (Figure 9b). This topological transformation confirms that the evolution mechanism effectively propagates knowledge from these seeds across the system, addressing the label sparsity challenge.

However, scale expansion must not compromise physical fidelity. To verify that the newly inferred relations are not merely semantic noise, we conducted a micro-level physical consistency analysis on the spatial distance distribution of relations inferred by the top 5 templates.

**Figure 10:**
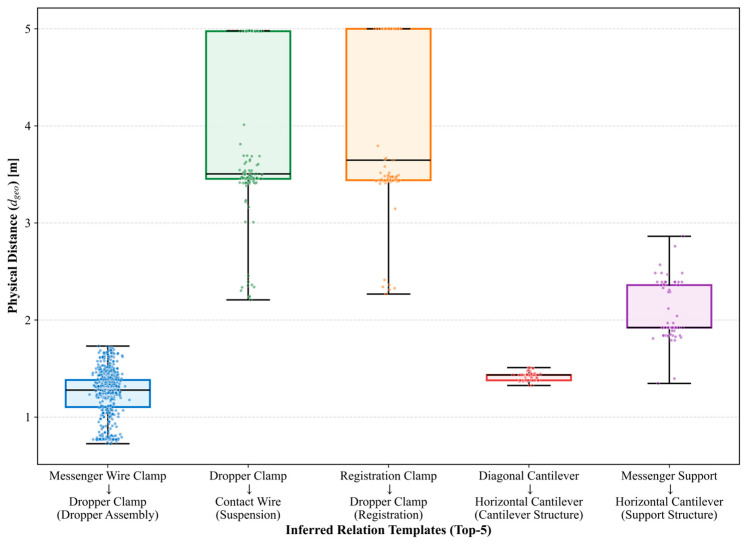
Physical consistency evaluation of inferred relations.

As visualized in Figure 10, the inferred relations exhibit high statistical compactness, validating that the evolution mechanism captures stable geometric constraint knowledge rather than performing random prediction. Specifically, we observe two distinct physical patterns. First, regarding rigid stability, for rigid metallic connections such as the cantilever structure (*diagonal cantilever* → *horizontal cantilever*), the distribution is extremely concentrated (σ=0.09 m), indicating the algorithm’s ability to lock onto precise geometric assemblies. Second, regarding flexible bounding, for flexible components like the dropper assembly (Rank 1, N=797), the lengths are distributed within a reasonable physical envelope (mean=1.22 m, range [0.60, 1.73] m). This accurately reflects the variable length of droppers required to maintain the contact wire’s level across the span.

These quantitative results confirm that PhyGeo-KG achieves massive scale growth while strictly adhering to the rigorous physical laws encoded in the multi-dimensional ontology.

#### 5.4.2. Case Study: Reasoning-Driven Semantic Indexing for Semantic Digital Twins

**Figure 11:**
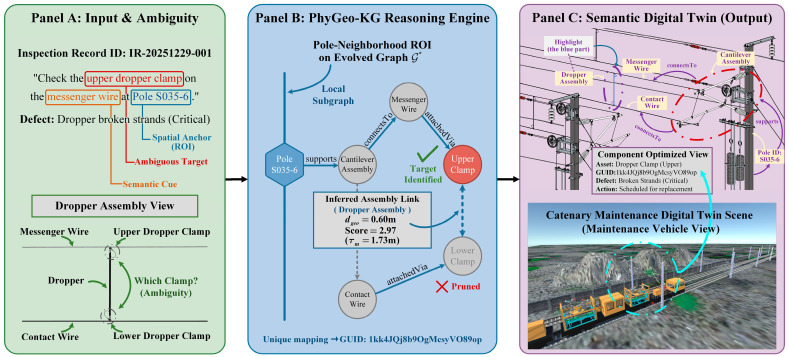
Reasoning-driven semantic indexing for semantic digital twins. (**Panel A**) Input and Ambiguity: Inspection instruction containing semantic ambiguity (“*upper clamp*”) and a spatial anchor (*Pole S035-6*). (**Panel B**) PhyGeo-KG Reasoning Engine: Reasoning path within the pole neighborhood ROI on the evolved graph $G^{*}$. The exported graph stores physically feasible neighborhood links as generic *related_to* edges with geometric distance and confidence scores (e.g., $d_{\mathrm{geo}}=0.60$ m, completion score=2.97, under $\tau_{m}=1.73$ m). The displayed labels (e.g., *supports*, *attachedVia*) denote an ontology-interpreted semantic view derived from completion templates for readability. (**Panel C**) Semantic Digital Twin Output: The uniquely indexed upper dropper clamp is highlighted in the BIM scene with defect attributes overlaid.

To further illustrate the practical value of PhyGeo-KG in real-world digital twin applications, we present a semantic indexing case study using a realistic inspection scenario. This capability exemplifies the SDT paradigm, transforming a static BIM model into a reasoning-enabled cognitive entity capable of interpreting maintenance records for automated, instance-level fault localization. The complete workflow of this paradigm is visualized in Figure 11.

Scenario and ambiguity challenge. Consider a typical inspection record derived from the Catenary-KG-2K dataset (Figure 11, Panel A):

Maintenance Record Extract:

Instruction: *Check the upper dropper clamp on the messenger wire at Pole S035-6 (K18 + 718)*.

Defect Type: *Dropper broken strands (Level 1, critical)*.

Even with an explicit pole anchor (S035-6), achieving instance-level indexing remains challenging due to extreme component repetition. Our dataset contains 824 messenger-wire dropper clamps and 835 contact-wire dropper clamps (1659 in total), most of which are geometrically similar. In the raw IFC model, the upper clamp (messenger-side) and lower clamp (contact-side) are isolated geometric entities without explicit functional associations. Traditional keyword-based retrieval therefore fails to reliably distinguish upper from lower, resulting in high ambiguity.

PhyGeo-KG solution: ontology-guided reasoning. PhyGeo-KG resolves the ambiguity by reasoning over the evolved graph $G^{*}$ within a pole/span-neighborhood region of interest (ROI) induced by the pole anchor, as detailed in the reasoning engine workflow (Figure 11, Panel B). The process follows two distinct steps:

1. Entity extraction and ROI pruning. The semantic sub-ontology extracts the target asset (“*upper dropper clamp*”) and location anchor (“*Pole S035-6*”). Using the deterministic grounding strategy (Section 4.2), the search is restricted to the local subgraph of the pole neighborhood. This local ROI acts as a spatial boundary, effectively pruning hundreds of spatially irrelevant dropper clamps located on other spans.
2. Physics-constrained multi-hop reasoning. Within this ROI, the system traverses template-completed edges in $G^{*}$. Although exported edges are stored as generic adjacency predicates (*related_to*) with geometric distances and confidence scores, we interpret them here using ontology-derived semantic labels (e.g., *supports*, *attachedVia*) to illustrate engineering logic. Ontology-guided templates (such as the dropper assembly template) enforce physical adjacency constraints to logically link the messenger-side clamp to its contact-side counterpart. Because the geometric distance ($d_{\mathrm{geo}}=0.60$ m) satisfies the physical admissibility bound ($\tau_{m}=1.73$ m), the system successfully reconstructs the latent assembly structure, thereby distinguishing the “upper” clamp based on its verified structural context rather than relying on brittle keyword matching.

As shown in the completion output (Figure 11, Panel B), a representative messenger-side clamp (*GUID: 1kk4JQj8b9OgMcsyVO89op*) connects to its contact-side counterpart (*GUID: 1kk4JQj8b9OgMcsyVO89om*) with a geometric distance $d_{\mathrm{geo}}=0.60$ m and a completion score of 2.97 (under bound $\tau_{m}=1.73$ m). Since $d_{\mathrm{geo}}<\tau_{m}$, the adjacency is physically admissible. This verified link reconstructs the latent dropper assembly absent in raw BIM. Combined with the textual cue “messenger wire,” the system precisely disambiguates the target as the upper clamp.

Results and engineering impact. The query resolves to a unique BIM instance (e.g., *IfcProduct GlobalId (GUID): 1kk4JQj8b9OgMcsyVO89op*). As visualized in the semantic digital twin output (Figure 11, Panel C), the system automatically highlights the exact component and overlays defect attributes for intuitive fault localization.

This case study validates the ability of PhyGeo-KG to bridge ambiguous natural language and precise instance-level BIM localization by reconstructing missing engineering logic. It positions PhyGeo-KG as a cognitive backbone for semantic digital twins, enabling automated fault localization and visualization, and significantly reducing on-site verification time in critical infrastructure maintenance.

## 6. Discussion

This study aimed to overcome the semantic–physical disconnect in engineering knowledge graph construction by developing PhyGeo-KG, a physics-regularized distant supervision framework that unifies unstructured maintenance logs with structured BIM/IFC models for catenary maintenance. The framework substantially suppressed semantic hallucinations through physics-aware refinement, delivered high-precision and physically consistent relations via dual gating, and achieved substantial graph expansion while preserving compliance, thereby providing traceable instance-level grounding under cold-start conditions.

These outcomes are consistent with the white-box design philosophy adopted in this work. The Physics Critic, instantiated as an explicit geometry–topology locality solver, acts as a differentiable soft logic gate that penalizes long-distance semantic bias without requiring large labeled datasets. Our progressive filtering pipeline demonstrates that embedding computable physical-feasibility cues throughout the extraction loop effectively filters physically implausible edges while preserving semantically supported ones. This supports our hypothesis that physical constraints function as active regularizers rather than mere post hoc filters. The ontology-driven evolution mechanism further propagates high-confidence seeds into a dense network by enforcing the same Physics Critic on inferred triples, ensuring that logical completion remains bounded by geometric reality. This mechanistic integration explains why the refined core graph maintains interpretability and why newly added relations exhibit stable physical signatures.

The approach advances prior work in several respects. While distant supervision pipelines such as Snorkel [23] and subsequent denoising strategies [24,25] effectively handle semantic noise, they remain language-driven and are not designed to suppress geometrically infeasible relations. Similarly, geometry-informed KG construction from BIM or CAD repositories [15,17] typically treats geometric data as static metadata or post-processing checks, whereas PhyGeo-KG embeds explicit topological constraints as first-class regularizers from the candidate-generation stage onward. In the context of Semantic Digital Twins [1,14], the present white-box instantiation highlights a practical pathway for addressing cold-start and traceability challenges in safety-critical linear infrastructure under severe label sparsity.

Academically, PhyGeo-KG contributes a generalizable S–G–P–P ontology and a pluggable Physics Critic that can be instantiated with domain-specific solvers, offering a reproducible template for physics-regularized multimodal KG construction. The framework is transferable primarily at the architectural level. The S–G–P–P ontology pattern, deterministic grounding logic, and physics-regularized prune-and-evolve workflow can be reused across other infrastructure domains (e.g., bridges, pipelines). However, domain-specific adaptations are required at the instantiation layer: the semantic lexicon, IFace taxonomy, mechanism models (e.g., structural stress models for bridges instead of tension solvers), and procedural rules must be re-instantiated for each target system. Furthermore, the Physics Critic in PhyGeo-KG is designed as a pluggable mechanism layer. While the current study instantiates it as a computationally efficient geometry–topology locality solver suitable for static catenary maintenance, the framework can incorporate richer critics—such as PDE-based physical simulations or force equilibrium models—for other safety-critical applications. For large-scale KG construction, incorporating full PDE solvers would incur substantially higher computational costs. To address this, we envision a “two-tier cascading screening” strategy for future deployments: inexpensive geometric critics perform coarse pruning, while higher-fidelity mechanism critics are selectively invoked only for ambiguous or safety-critical candidates to balance computational efficiency and physical rigor. For engineering applications, the framework provides a knowledge substrate for Semantic Digital Twins, enabling automated instance-level fault localization and reasoning over maintenance records directly linked to BIM entities.

Several limitations should be acknowledged. First, the current instantiation targets BIM-available scenarios and excels in zero-shot cold-start conditions by relying on explicit ontology typing and local topology rather than labeled embeddings. However, when IFC-grounded geometry is unavailable or strongly incomplete (e.g., implicit components missing from the BIM geometry), the framework may retain text-side evidence but cannot construct a physics-verified core graph with the same degree of geometric traceability. Mentions that cannot be reliably grounded due to irregular colloquial names or missing BIM entities are conservatively excluded from the physics-verified core graph rather than being force-matched. Furthermore, when repeated components are locally near-symmetric and share highly similar geometric bounds within a single ROI, relying solely on static semantic–geometric adjacency constraints will yield overlapping confidence scores. In such cases of extreme local repetition, additional cues such as serial position, installation metadata, or temporal inspection context are required for reliable disambiguation.

In addition, the current implementation assumes static topology and therefore does not capture pantograph–catenary dynamic fluctuations, electrical properties, load conditions, or time-variant fault propagation. These constraints limit immediate extrapolation to real-time dynamic monitoring scenarios, although they do not affect the validity of the static-maintenance use cases demonstrated here.

## 7. Conclusions and Future Work

This work addressed the longstanding semantic–physical disconnect that has limited the construction of reliable engineering knowledge graphs for high-speed railway catenary maintenance. In this domain, unstructured maintenance records must be accurately linked to geometric BIM assets under severe label sparsity and stringent traceability requirements. To this end, we developed PhyGeo-KG, a physics-regularized distant supervision framework that embeds explicit physical feasibility as active, computable regularizers throughout the extraction and refinement process.

The framework integrates geometry-anchored deterministic grounding, physics-aware refinement via a pluggable Physics Critic, and ontology-driven evolution. It produces high-fidelity multimodal geometric knowledge graphs that are semantically meaningful, physically consistent, and fully traceable to precise BIM entities. Furthermore, it enables controlled, physically bounded graph expansion from a reliable refined core. This offers a practical and interpretable solution to the cold-start challenge in safety-critical engineering domains.

PhyGeo-KG advances both methodology and practice. Methodologically, it contributes a generalizable Semantic–Geometric–Physical–Procedural (S–G–P–P) ontology and a white-box Physics Critic that can serve as a reproducible template for physics-regularized knowledge graph construction in other label-scarce domains. In practice, it strengthens the foundation for Semantic Digital Twins by enabling direct asset-level linkage between unstructured maintenance records and geometric infrastructure models, thereby supporting reliable instance-level indexing and reasoning.

Building on the above findings and limitations, future work will extend PhyGeo-KG in three main directions. First, to address the limitations of the current static setting, the framework will be expanded toward sensor-integrated spatio-temporal physical knowledge graphs. By incorporating time-series condition-monitoring signals, vision-sensor imagery, and higher-fidelity PDE-based mechanism models (e.g., for electrical properties and dynamic tension), the framework aims to capture pantograph–catenary dynamic fluctuations and enable real-time semantic indexing, anomaly explanation, and predictive maintenance decision support. Second, downstream digital twin applications can be further developed, including automated repair simulation and virtual maintenance training, to better support real-world maintenance workflows. Third, the generalizability of the approach should be tested across other linear infrastructures, such as bridges and tunnels, by adapting the S–G–P–P ontology and physics critic to different domain constraints.

Overall, PhyGeo-KG demonstrates that embedding physical constraints as active regularizers can help bridge the semantic–geometric–physical gap in multimodal knowledge graph construction, providing a more trustworthy and traceable foundation for digital twin-enabled maintenance in critical infrastructure.

## Figures and Tables

**Table 1:** Summary of key notations and symbols.

Category	Symbol	Description
Input and Graph States	$D_{\mathrm{geo}},D_{\mathrm{txt}}$	The raw inputs: IFC-compliant BIM models and unstructured maintenance texts (logs, regulations, manuals).
	$G^{ifc}$	The Native IFC Graph parsed from BIM models, preserving topological aggregation.
	$G_{\Omega (m)}^{ifc}$	The Local IFC Subgraph extracted within the spatial region of interest Ω(m).
	$G^{\mathrm{txt}}$	The Text-Side Virtual Graph representing mentions and syntactic relations parsed from unstructured logs.
	$G_{0}$	The Initial Candidate Graph (high-recall) generated via deterministic grounding.
	$G_{\mathrm{core}}^{*}$	The Refined Core Graph containing only physics-verified, high-confidence relations.
	$G^{*}$	$\mathrm{The}\;\mathrm{Final}\;\mathrm{Evolved}\;\mathrm{Graph}\;\mathrm{expanded}\;\mathrm{from}\;G_{\mathrm{core}}^{*}$ via ontology-driven inference.
Ontology and Anchors	O	$\mathrm{The}\;\mathrm{multi}-\mathrm{dimensional}\;S–G–P–P\;\mathrm{ontology}\;(O^{\mathrm{sem}},O^{\mathrm{geo}},O^{\mathrm{phy}},O^{\mathrm{pro}}$).
	IFace(i)	$\mathrm{The}\;\mathrm{Geometric}\;\mathrm{Interface}\;\mathrm{anchor}\;\mathrm{defined}\;\mathrm{by}\;(c_{i},n_{i},\tau_{i},s_{i})$.
	m,a	Text mention m and its aligned geometric anchor a (IFC entity or IFace).
	Ω(m)	The Region of Interest (ROI) induced by location cues for spatial pruning.
Model and Physics	e=(u,v)	A candidate relation edge connecting entities u and v.
	$s_{e}$	$\mathrm{The}\;\mathrm{Semantic}\;\mathrm{Confidence}\;\mathrm{Score}\;(s_{e}\in (0,1)$) learned by the Evidence Aggregator.
	$c_{e}$	$\mathrm{The}\;\mathrm{Physical}\;\mathrm{Plausibility}\;\mathrm{Score}\;(c_{e}\in (0,1]$) computed by the Physics Critic.
	$v_{\mathrm{phy}}(e)$	$\mathrm{The}\;\mathrm{Physics}\;\mathrm{Violation}\;\mathrm{penalty}\;\mathrm{term}\;(v_{\mathrm{phy}}(e)=1-c_{e}$).
	$d_{\mathrm{geo}}(a_{u},a_{v})$	$\mathrm{The}\;\mathrm{Euclidean}\;\mathrm{distance}\;\mathrm{between}\;\mathrm{the}\;\mathrm{IFace}\;\mathrm{centroids}\;\mathrm{of}\;\mathrm{anchors}\;a_{u}$$\;\mathrm{and}\;a_{v}$.
Constraints and Parameters	Γ	$\mathrm{The}\;\mathrm{hierarchical}\;\mathrm{constraint}\;\mathrm{set}\;(\Gamma_{\mathrm{basic}}\cup \Gamma_{\mathrm{full}}$) used for legality pruning and physics regularization.
	$d_{0}$	The Characteristic Interaction Scale (threshold for physical regularization).
	$\tau_{\mathrm{sem}},\tau_{\mathrm{phy}}$	The Dual Gating Thresholds for semantic acceptance and physical plausibility.

**Table 2:** Representative classes, relations, and computable hooks in the ontology.

Sub-Ontology	Key Classes (Examples)	Key Relations	Computable Hooks (White-Box Instantiation)
$O^{\mathrm{sem}}$ (Semantic)	*Asset*, *Defect*, *Location*, *FunctionRole* (e.g., *Dropper*, *FatigueFracture*, *Span*, *supporting*)	*hasDefectType*; *locatedAtSpan*; *hasFunctionRole*	Lexicon Matcher: mention → canonical URI via synonym dictionary (e.g., “broken strand” → *FatigueFracture*). Pattern Typer: rules to type phrases (e.g., “M16 bolt” → *Resource*/*Tool*).
$O^{\mathrm{geo}}$ (Geometric)	*IfcProduct*, *IFace* (e.g., *BoltHole*, *ClampSurface*)	*hasIFace*; *spatiallyContains*; *connectedTo*	$\mathrm{IFace}\;\mathrm{Parser}:\;\mathrm{extracts}\;\mathrm{IFace}(i)=(c_{i},n_{i},\tau_{i},s_{i})$ from IFC B-Rep and anchors it to IFC instances. $\mathrm{ROI}\;\mathrm{Filter}:\;\mathrm{computes}\;\Omega (m)\;\mathrm{from}\;\mathrm{location}\;\mathrm{cues}\;\mathrm{and}\;\mathrm{extracts}\;\mathrm{local}\;\mathrm{subgraph}\;G_{\Omega (m)}^{ifc}$.
$O^{\mathrm{phy}}$ (Physical)	*PhysicalQuantity*, *MechanismModel* (e.g., *Tension*, *Sag*, *Stiffness*)	*parameterizesModel*; *violatesConstraint*	$\mathrm{Interaction}\;\mathrm{Critic}:\;c_{e}=\exp(-d_{\mathrm{geo}}/d_{0})$$,\;v_{\mathrm{phy}}(e)=1-c_{e}$. $\mathrm{Catenary}\;\mathrm{Solver}\;(\mathrm{example}):\;y(x)=\frac{T}{w}\cosh(\frac{w}{T}x)-\frac{T}{w}$.
$O^{\mathrm{pro}}$ (Procedural)	*MaintenanceTask*, *Resource*, *MaintenanceStep* (e.g., *Adjustment*, *TorqueWrench*)	*requiresTool*; *performedBy*; *precededBy*	Logic Checker: verifies procedural compliance (e.g., *requiresTool* satisfied in extracted relations). Precondition Validator: checks step inputs match resource definitions.

**Table 3:** Taxonomy of hierarchical constraints and their stage-specific usage.

Constraint Type	Category	Nature	Representative Examples/Logic	Primary Usage Stage	Enforcement
Hard $(\Gamma_{\mathrm{basic}}$)	Domain/Range Validity	Boolean	type(u)∈Dom(r) ∧ type(v)∈Rng(r)	Candidate generation (Section 4.2)	Strict filtering and rejection
	IFC Structural Legality	Boolean	$\mathrm{Valid}\;\mathrm{aggregation}/\mathrm{containment}/\mathrm{connectivity}\;\mathrm{path}\;\mathrm{in}\;G^{ifc}$ (e.g., *IfcRelAggregates*, *IfcRelContainedInSpatialStructure*, *IfcRelConnectsElements*)	Candidate generation (Section 4.2)	Native IFC schema validation
	Spatial ROI Pruning	Boolean	Anchor a∈Ω(m) induced by location cues	Candidate generation (Section 4.2)	ROI-based subgraph extraction
	Topological Signature Matching	Boolean	k-hop neighborhood compatibility/topology-similarity thresholding	Candidate generation (Section 4.2)	Deterministic pruning (topology check)
Soft $(\Gamma_{\mathrm{full}}$)	Geometric Distance	Differentiable	$c_{e}=\exp(-d_{\mathrm{geo}}/d_{0})$$,\;v_{\mathrm{phy}}(e)=1-c_{e}$ (Equation (8))	Physics-regularized refinement (Section 4.3) Physics-regularized refinement (Section 4.3) Physics-regularized refinement (Section 4.3)	Physics Critic penalty term
	Physical Plausibility	Differentiable/graded	Mechanism-model residual as graded violation (e.g., catenary sag equilibrium within tolerance)		$L_{\mathrm{phy}}$ regularization
	Procedural Compliance	Rule-based/semi-soft	*requiresTool* satisfied; step preconditions met		Logic checker + constraint penalty
	Logical Consistency	Logical/graded	Cardinality restrictions; inverse/transitive property consistency	Refinement and evolution validation (Section 4.3.5)	Post hoc logic filtering and template validation

Note: Hard constraints ($\Gamma_{\mathrm{basic}}$) are enforced deterministically during candidate graph generation to guarantee schema legality and maintain high recall under cold-start settings. Soft constraints ($\Gamma_{\mathrm{full}}$) serve as differentiable and/or graded regularizers in physics-aware refinement, enabling the Physics critic and dual gating while preserving interpretability and engineering traceability.

**Table 4:** Schema definition of the text-side virtual graph ($G^{\mathrm{txt}}$) and alignment constraints.

Graph Element	Type/Category	Attributes and Provenance	Grounding Operation (Link to BIM)
$\mathrm{Nodes}\;(V^{\mathrm{txt}}$)	Asset/Component (e.g., *Dropper*, *Pole*, *Clamp*)	$\mathrm{MentionSpan},\;\mathrm{SurfaceForm},\;\mathrm{EntityType}\;(\in O^{\mathrm{sem}}$); Traceability: *DocID*, *SentID*, *TokenSpan*	Deterministic Matching: (1) Filter: a∈Ω(m)$\;\mathrm{and}\;I_{\mathrm{type}}(m,a)=1$$\;(\mathrm{ontology}-\mathrm{consistent}\;\mathrm{type}\;\mathrm{match})\;(\Gamma_{\mathrm{basic}})$ (2) Rank: compute S(m,a) and keep TopK(m) anchors.
	Defect/State (e.g., *Fracture*, *Loose*)	*DefectType*, *SeverityLevel* (*optional*), *TriggerWord*; Traceability: *DocID*, *SentID*, *TokenSpan*	$\mathrm{Indirect}\;\mathrm{Anchoring}:\;\mathrm{attached}\;\mathrm{to}\;\mathrm{its}\;\mathrm{associated}\;\mathrm{asset}\;\mathrm{mention};\;\mathrm{inherits}\;\mathrm{the}\;\mathrm{asset}’s\;\mathrm{candidate}\;\mathrm{anchors}\;\mathrm{TopK}(m_{asset})$ for subsequent refinement.
	Location Cue (e.g., “*Span 135*”, “*Pole S035-6*”)	*LocType* (*SpanID*/*PoleID*/*KmMark*), *LocValue*; Traceability: *DocID*, *SentID*, *TokenSpan*	ROI Induction: constructs Ω(m)$\;\mathrm{to}\;\mathrm{extract}\;\mathrm{local}\;\mathrm{subgraph}\;G_{\Omega (m)}^{ifc}$ and prune the search space.
$\mathrm{Edges}\;(E^{\mathrm{txt}}$)	Syntactic Dependency (e.g., *nsubj*, *dobj*)	*DepLabel*, *TokenDistance*, *DependencyPath*; Traceability: *DocID*, *SentID*	Internal Feature: used for pattern extraction; not directly mapped.
		RelationType (∈O), *Pattern*/*TriggerSpan*, *Confidence* (*optional*); Traceability: *DocID*, *SentID*	$\mathrm{Candidate}\;\mathrm{Lifting}:\;\mathrm{instantiates}\;\mathrm{candidate}\;\mathrm{triple}\;(a_{u},r,a_{v})\in E^{\mathrm{cand}}$$\;(\mathrm{thus}\;\mathrm{forming}\;G_{0})$ once both endpoints are grounded.
	Semantic Relation (e.g., *hasDefectType*)		

Note: Notations follow Table 1. Ω(m) denotes the ROI induced by location cues, S(m,a) is the deterministic mention–anchor alignment score, and TopK(m) is the top-K anchor set used for candidate lifting in Section 4.2.3.

**Table 5:** Design of representative labeling functions (LFs) for weak supervision.

LF ID	Function Type	Trigger Condition/Rule Logic	Vote	Design Intent (Typical Scope)	Corresponding Constraint/Source
LF1	Syntactic Pattern	Dependency path matches relation-specific SVO templates (e.g., *Asset*–*Verb*–*Asset*, *Asset*–*hasDefect*–*Defect*)	+1	High-Recall Heuristic (High coverage)	Text heuristic (dependency patterns)
LF2	Keyword Co-occurrence	Relation-specific keywords co-occur within context window w (e.g., “*fracture*” near “*dropper*”)	+1	Domain Generalist (Medium coverage)	$O^{\mathrm{sem}}$ lexicon (domain knowledge)
LF3	Schema Violation	Domain/range typing violated (e.g., Defect linked to Location under incompatible relation)	−1	Safety Net (negative vote) (Low coverage)	$\Gamma_{\mathrm{basic}}$ schema validity (type check)
LF4	Spatial Mismatch	Mention indicates “Span X” but anchor falls in “Span Y” (cross-span mismatch)	−1	Contextual Contradiction (Medium coverage)	$\Gamma_{\mathrm{basic}}$ spatial locality (ROI constraint)
LF5	IFace Incompatibility	Anchor lacks required IFace implied by text (e.g., fastener relation but no compatible interface)	−1	Fine-Grained Contradiction (Low coverage)	$O^{\mathrm{geo}}$ $\;\mathrm{IFace}\;\mathrm{hook}+\Gamma_{\mathrm{basic}}$
LF6	Geometric Feasibility	$\mathrm{Over}-\mathrm{distance}:\;d_{\mathrm{geo}}(a_{u},a_{v})>\alpha d_{0}$ (e.g., α∈[2,3])	−1	Physics Prior (negative vote) (Medium coverage)	$\mathrm{Geometry}-\mathrm{based}\;\mathrm{heuristic}\;(d_{\mathrm{geo}}$$\;\mathrm{vs}.\;d_{0}$)
LF7	Procedural Compliance	Procedural trigger present but required tool/resource evidence missing	−1	Logic Consistency Check (Low coverage)	$O^{\mathrm{pro}}$ compliance (procedural constraint)
LF8	High-Precision Combo	$\mathrm{Strong}\;\mathrm{syntactic}\;\mathrm{pattern}\;\mathrm{and}\;\mathrm{close}\;\mathrm{proximity}:\;d_{\mathrm{geo}}<\kappa d_{0}$ (e.g., κ=0.5)	+1	High-Precision Specialist (Low coverage)	Multi-signal (semantic + geometric)

Note: Each LF outputs a discrete vote in {−1,0,+1}. Votes are aggregated by a generative label model (majority vote as a simple baseline) to produce probabilistic weak labels ${\hat{y}}_{e}\in [0,1]$ for distant supervision. Unlike deterministic pruning in Section 4.2, negative votes here provide discriminative evidence for probabilistic refinement rather than hard rejection. Candidate edges not triggered by any LF receive an abstention vote (0) by default.

**Table 6:** Statistics of the Catenary-KG-2K dataset.

Data Category	Item	Count	Description
$\mathrm{Geometry}\;(D_{\mathrm{geo}}$)	IFC Entities	6861	Extracted from .ifc files (with 100% IFace generation coverage)
$\mathrm{Text}\;(D_{\mathrm{txt}}$)	Sentences	8628	Segmented from 350 maintenance documents
	Mentions	17,746	Domain entities (Assets, Defects, Locations)
$\mathrm{Graph}\;(G_{raw}$)	Candidate Edges	3238	Initial co-occurrence relations (Text-only)

**Table 7:** Performance comparison of graph construction stages.

Method	Precision@50	PCR	${\bar{d}}_{\mathrm{geo}}$ (m)	Subset Precision	Subset Recall	Subset F1-Score
Text-only (baseline)	0.56	34%	19.651	-	-	-
Geo-aware (baseline)	0.82	54%	6.048	0.67	1.00	0.80
PhyGeo-KG (core)	0.90	86%	3.366	0.90	0.94	0.92

Note: (1) The “Text-only” baseline operates on ungrounded textual mentions rather than explicitly localized BIM instances. Therefore, Subset Recall and F1-score are not applicable to this baseline, as these metrics are strictly evaluated on the geometrically grounded subset to assess the Physics Critic’s trade-off. (2) ${\bar{d}}_{geo}$ denotes the average value of $d_{geo}$, serving as a proxy for physical compactness.

**Table 8:** Sensitivity analysis of the characteristic interaction scale ($d_{0}$).

$d_{0}$ (m)	Retained Edges	Subset Precision	Subset Recall	Subset F1-Score	PCR	HRR
10	57	0.96	0.69	0.80	89%	94.4%
15	72	0.94	0.85	0.89	88%	86.8%
20	83	0.90	0.94	0.92	86%	79.4%
25	96	0.81	0.98	0.89	80%	61.2%
30	107	0.74	0.99	0.84	73%	45.2%

Note: Subset Precision, Subset Recall, and Subset F1-score are evaluated on the fully annotated subset of 120 geometrically grounded candidate edges to rigorously track the trade-off of the Physics Critic.

## Data Availability

The datasets generated during and/or analyzed during the current study are available from the corresponding author on reasonable request.
